# Evolution of Chemical Diversity in a Group of Non-Reduced Polyketide Gene Clusters: Using Phylogenetics to Inform the Search for Novel Fungal Natural Products

**DOI:** 10.3390/toxins7093572

**Published:** 2015-09-10

**Authors:** Kurt Throckmorton, Philipp Wiemann, Nancy P. Keller

**Affiliations:** 1 Department of Genetics, University of Wisconsin-Madison, 425 Henry Mall, Madison, WI 53706-1580, USA; E-Mail: kthrockmorto@wisc.edu; 2 Department of Medical Microbiology and Immunology, University of Wisconsin-Madison, 1550 Linden Drive, Madison, WI 53706-1521, USA; E-Mail: pwiemann@wisc.edu

**Keywords:** mycotoxin, gene cluster, evolution, polyketide synthase

## Abstract

Fungal polyketides are a diverse class of natural products, or secondary metabolites (SMs), with a wide range of bioactivities often associated with toxicity. Here, we focus on a group of non-reducing polyketide synthases (NR-PKSs) in the fungal phylum Ascomycota that lack a thioesterase domain for product release, group V. Although widespread in ascomycete taxa, this group of NR-PKSs is notably absent in the mycotoxigenic genus *Fusarium* and, surprisingly, found in genera not known for their secondary metabolite production (e.g., the mycorrhizal genus *Oidiodendron*, the powdery mildew genus *Blumeria*, and the causative agent of white-nose syndrome in bats, *Pseudogymnoascus destructans*). This group of NR-PKSs, in association with the other enzymes encoded by their gene clusters, produces a variety of different chemical classes including naphthacenediones, anthraquinones, benzophenones, grisandienes, and diphenyl ethers. We discuss the modification of and transitions between these chemical classes, the requisite enzymes, and the evolution of the SM gene clusters that encode them. Integrating this information, we predict the likely products of related but uncharacterized SM clusters, and we speculate upon the utility of these classes of SMs as virulence factors or chemical defenses to various plant, animal, and insect pathogens, as well as mutualistic fungi.

## 1. Introduction

The Kingdom Fungi is well known for its ability to synthesize bioactive secondary metabolites (SMs), also known as natural products. The dominant taxa producing SMs belong to several filamentous ascomycete genera, many of which are renowned plant, insect, and/or human pathogens. Virulence is often associated with the production of toxic SMs in these fungi [1]. In nature, where studied, SMs afford various fitness advantages to the producing species ranging from protection from fungivory and physical insults (e.g., UV light) to competition with other microbes for niche securement [2,3,4].

SMs can be classified according to chemical types. For example, polyketides are derived from acetyl/malonyl-coenzyme A (CoA), non-ribosomal peptides from amino acids, and terpenes from isoprene. The reader is referred to several recent reviews for in-depth coverage of each chemical class [5,6,7]. Each class is associated with a defining synthase, polyketide synthase (PKS), non-ribosomal peptide synthetase (NRPS), and terpene cyclase/synthase, respectively. Ribosomal peptides [8] and hybrid synthases, commonly hybrid PKS-NRPS enzymes, have also been described. The synthases contain conserved catalytic domains easily detectable by statistical analysis, *i.e.*, hidden Markov models, thus making them highly suitable for phylogenetic analyses and evolutionary inferences [9,10,11]. Typically, the genes encoding these synthases are physically clustered with additional enzymatic genes required to form the end metabolite; these are termed SM gene clusters.

Polyketides in particular have drawn considerable interest due to their impact on human and plant health both positively (e.g., lovastatin) and detrimentally (e.g., aflatoxin). The first fungal PKS to be identified and characterized was 6-methylsalicylic acid synthase, found in several *Penicillium* and *Aspergillus* spp. [12,13,14]. Shortly thereafter, PKSs required for spore pigmentation as well as the mycotoxins aflatoxin and sterigmatocystin were characterized in several *Aspergillus* spp. [15,16,17,18]. Additional PKSs involved in toxin synthesis include Pks1 and Pks2 (T toxin in *Cochliobolus heterostrophus*, [19,20]), FusA (fusarin production in *Fusarium fujikuroi*, [21] and *F. verticillioides*, *F. venenatum* [22]), Zea1 and Zea2 (zearalenone production in *F. graminearum*, [23,24]), Fum1 (fumonisin production in *F. verticillioides*, *F. fujikuroi*, [25,26]), Pks-CT (citrinin production in *Monascus purpureus*, [27]), and NhPKS1 (bostrycoidin and fusarubin in *F. solani*, [28]). PKSs and hybrid PKS-NRPSs have also been associated with the development of fungal spores and overwintering structures such as sclerotia [29,30,31].

**Figure 1:**
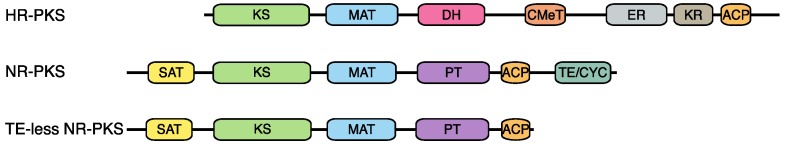
A diagram showing the domains of typical highly reducing (HR), non-reducing (NR), and group V NR type I fungal polyketide synthases. SAT = starter-unit:ACP transacylase, KS = β-ketoacyl synthase, MAT = malonyl-CoA:ACP transacylase, DH = dehydratase, CMeT = *C*-methyltransferase, ER = enoyl reductase, KR = ketoreductase, PT = product template, ACP = acyl carrier protein, TE = thioesterase, CYC = Claisen cyclase.

Most fungal PKSs are multi-functional enzymes known as iterative type I PKSs, where each catalytic domain is encoded in one gene, though a few fungal PKSs are of type III [32]. There are two main classes of type I PKS known as non-reducing (NR) and highly reducing (HR). All PKSs harbor three essential domains—the β-ketoacyl synthase (KS), malonyl-CoA:acyl carrier protein transacylase (MAT), and acyl carrier protein (ACP) domains—however, NR- and HR-PKSs vary in their catalytic domains that impact the reduction or dehydration steps of the growing carbon chain (Figure 1). The minimal architecture of HR-PKSs is typically composed of ketoreductase (KR), dehydratase (DH), and enoyl reductase (ER) domains, thereby resembling fatty acid synthases (FASs). Another key difference is the presence of a *C*-methyltransferase (CMeT) domain found in most HR-PKSs and only in one subset of NR-PKSs (e.g., *A. nidulans* AfoE, [33]). However, despite the presence of CMeT domains in HR-PKSs, analysis in *Fusarium* spp. suggests that the domain can be non-functional [34].

NR-PKSs are characterized by the absence of KR, DH, and ER domains and the presence of starter-unit:ACP transacylase (SAT) and product template (PT) domains. While N-terminal SAT domains of most NR-PKSs accept acetyl-CoA as starter unit [35] there are certain examples where the starter unit is either provided by dedicated FAS-like proteins [36] or HR-PKSs [37] which are usually encoded by genes located within the co-regulated cluster region. While the KS domain largely controls chain length [38], the PT domain, which is always located between the MAT and ACP domains, determines the cyclization pattern of the polyketide product [39]. The generated cyclized products of NR-PKSs are released by a variety of mechanisms, that in some cases contribute to cyclization patterns as well [40]. The thioesterase (TE)-mediated product release by a canonical TE domain is the most common mechanism (e.g., orsellinic acid/F9775 [41,42,43]). This domain often extends to a domain capable of C–C Claisen cyclization (a TE/CYC domain) (e.g., aflatoxin and sterigmatocystin PKSs and many pigment PKSs, [44,45]). In some cases, NR-PKSs contain a reductase-releasing (R) domain (e.g., asperfuranone [33]); in others, e.g., the PKS involved in asperthecin biosynthesis in *A. nidulans*, there is no releasing domain. These latter NR-PKSs are usually coupled with metallo-β-lactamase-type TE proteins (MβL-TE) that allow for release of the nascent polyketide chain. These MβL-TEs are encoded by distinct genes located within their respective gene clusters, as illustrated for asperthecin (AptB), endocrocin (EncB), viridicatumtoxin (VrtG), TAN-1612 (AdaB), geodin (GedB), monodictyphenone (MdpF), pestheic acid (PtaB), trypacidin (TpcB), and neosartoricin (NscB) [46,47,48,49,50,51,52,53]. Here we conducted a phylogenetic analysis of PKS genes from currently available fungal genomes, with a focus on the TE-less NR-PKSs, group V. Our data strengthen predictions of subdivisions within this group, and, moreover, we expand upon the predictive power of this analysis to suggest directions for future research.

## 2. Group V NR-PKS Phylogeny

**Figure 2:**
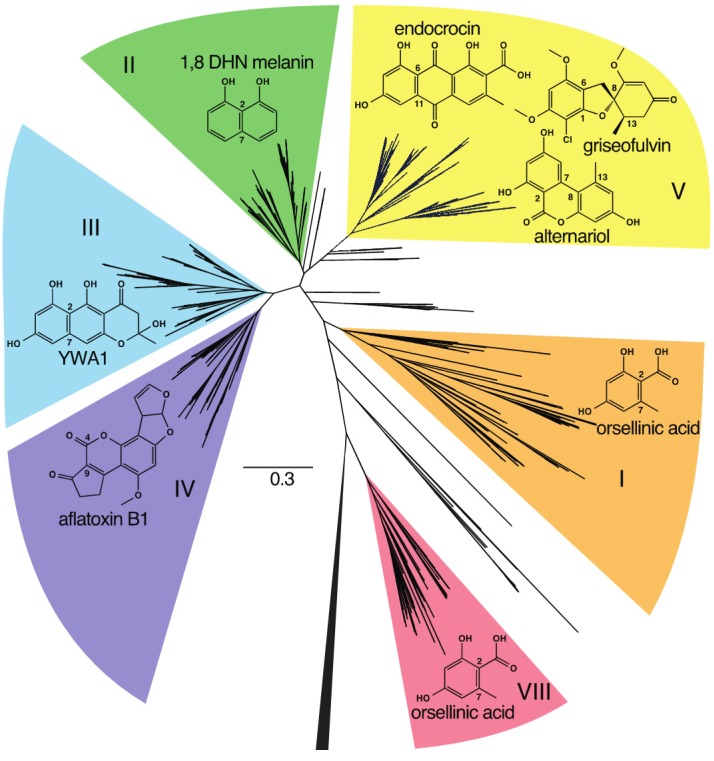
A maximum likelihood phylogenetic tree constructed with FastTree [60] using the KS domains of 908 NR-PKSs. Clades corresponding to characterized groups I–VIII are highlighted and labeled. No members of groups VI and VII were identified by our methods. Examples of structures produced by each group are shown in their respective highlighted regions and the numbering of the carbon-carbon bonds indicates the mode of cyclization of the PKSs that produce these compounds. The outgroup, consisting of the KS domains of 70 HR- and hybrid PKS/NRPSs, as well as the human fatty acid synthase, FASN, is collapsed.

**Figure 3:**
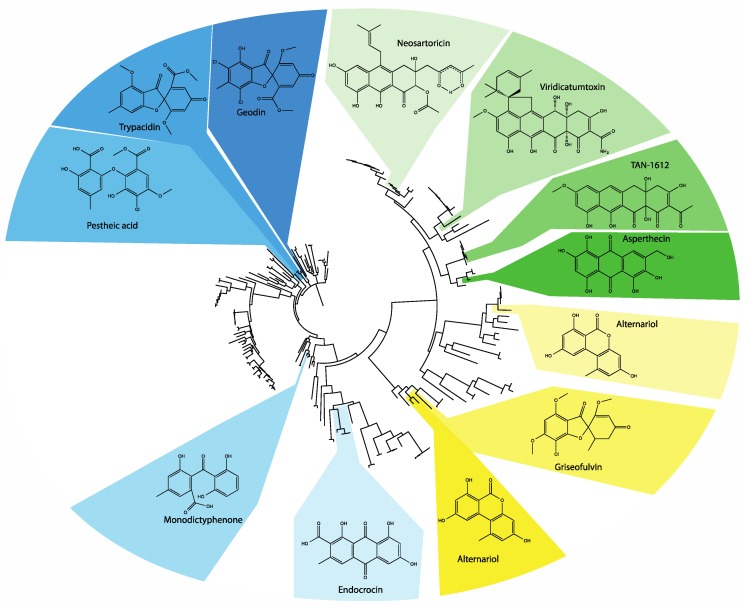
A maximum likelihood phylogenetic tree constructed with FastTree [60] using the KS domains of a subset of 188 group V NR-PKSs extracted from the broader set used above. The leaves corresponding to characterized PKSs are highlighted in shades of blue for group V1, green for group V2, and yellow for group V3. The structures produced by these PKSs and their associated decorating enzymes are shown adjacently.

Previous phylogenetic analyses have used the whole PKS, the KS domain, or the PT domain for comparison, but these have been noted to reflect one another, indicating their coevolution [54]. These studies classified the NR-PKSs first into three subclades [55], then further into five [56], seven [54], and most recently eight groups [57]. These NR-PKSs are present in many ascomycetes and some basidiomycetes, though only members of group VIII have been identified in basidiomycetes [57] with only one characterized example to date [58]. These groups generally represent unique combinations of product length and cyclization register. Of the eight current groups, we focus on NR-PKSs belonging to the TE-less group V in this study (Figure 2). Group V has thirteen characterized gene clusters with eleven described products including endocrocin, monodictyphenone, trypacidin, geodin, pestheic acid, asperthecin, TAN-1612, neosartoricin, viridicatumtoxin, griseofulvin, and alternariol (Table 1, Figure 3). All group V PKSs lack a domain for product release, but they vary in the length (hepta- to decaketide) of their products; most make C6–C11 connections, but a couple of exceptional cases make C1–C6 or C2–C7 connections. Generally, the thioesterase activity is encoded in a separate but adjacent MβL-TE. Based on our analysis of the KS domains of 908 fungal PKSs identified through the NCBI’s BLAST utility and manually using AspGD (Figure S1), we have identified 188 PKSs belonging to group V (Figure S2). These PKSs are derived from 88 species of 39 genera distributed across five classes of ascomycetes (Table 1). Notably, no group V PKSs were identified in the well characterized *Fusarium* spp. [59]. This phylogenetic tree allowed us to visualize relationships between these unknown PKSs and the thirteen examples, thus defining the best contexts in which to deduce the functions of the putative clusters to which these PKSs belong.

**Table 1:** List of the classes, genera, and species with PKSs belonging to group V, their accession numbers, their subgroup, characterized products, and predicted products. These predictions are based on the presence or absence of homologs of the requisite genes to produce these compounds as determined by previous studies of group V SM gene clusters. These requisite genes are discussed in the sections corresponding to either the relevant group (e.g., V1, V2, and V3) or compound (e.g., alternariol or viridicatumtoxin).

Class	Genus	Species	PKS	Sub-group	Characterized product	Predicted product	Reference
Dothidiomycete	*Alternaria*	*alternata*	AFN68300	V3	Alternariol	Characterized	[61]
	*Aureobasidium*	*melanogenum*	KEQ66138	V1	None	Emodin anthrone	This study
	*Bipolaris*	*maydis*	EMD89515	V3	None	No data	This study
			ENH99769	V3	None	No data	This study
			ENI07798	V1	None	Other anthrone	This study
			AAR90273	V1	None	No data	This study
			AAR90274	V3	None	No data	This study
		*oryzae*	EUC41199	V1	None	Benzophenone	This study
			EUC45162	V1	None	Other anthrone	This study
		*sorokiniana*	EMD62925	V1	None	Other anthrone	This study
			EMD66882	V1	None	Benzophenone	This study
		*victoriae*	EUN25734	V1	None	Other anthrone	This study
		*zeicola*	EUC29913	V1	None	Other anthrone	This study
	*Macrophomina*	*phaseolina*	EKG11397	V1	None	No product	This study
			EKG18431	V1	None	No data	This study
	*Phaeosphaeria*	*nodorum*	EAT76667	V3	Alternariol	Characterized	[62]
	*Pseudocercospora*	*fijiensis*	EME79056	V1	None	Emodin	This study
	*Pseudogymnoascus*	*destructans*	ELR08155	V1	None	Endocrocin anthrone	This study
		*pannorum*	KFY01830	V1	None	Endocrocin anthrone	This study
			KFY04191	V1	None	Other anthrone	This study
			KFY04767	V1	None	Endocrocin anthrone	This study
			KFY14212	V1	None	Emodin anthrone	This study
			KFY24933	V1	None	Endocrocin anthrone	This study
			KFY28347	V1	None	Endocrocin anthrone	This study
			KFY28376	V1	None	Endocrocin anthrone	This study
			KFY41668	V1	None	Emodin anthrone	This study
			KFY50141	V1	None	Grisandiene	This study
			KFY61750	V1	None	Endocrocin anthrone	This study
			KFY73941	V1	None	Endocrocin anthrone	This study
			KFY81274	V1	None	Other anthrone	This study
			KFY90954	V1	None	Other anthrone	This study
			KFY97098	V1	None	Endocrocin anthrone	This study
			KFZ02364	V1	None	Endocrocin anthrone	This study
			KFZ03027	V1	None	Other anthrone	This study
			KFZ03785	V1	None	Endocrocin anthrone	This study
			KFZ09857	V1	None	Endocrocin anthrone	This study
	*Pyrenophora*	*teres*	EFQ95560	V1	None	Endocrocin anthrone	This study
		*tritici*	EDU45231	V1	None	Endocrocin anthrone	This study
	*Setosphaeria*	*turcica*	EOA88807	V1	None	Other anthrone	This study
	*Sphaerulina*	*musiva*	EMF17386	V1	None	Other anthrone	This study
	*Verruconis*	*gallopava*	KIW05310	V1	None	No product	This study
Eurotiomycete	*Arthroderma*	*benhamiae*	EFE32713	V2	None	Neosartoricin, fumicycline	This study
		*otae*	EEQ30779	V2	None	Neosartoricin, fumicycline	This study
			EEQ31623	V3	None	Alternariol	This study
	*Aspergillus*	*acidus*	Aspfo1_0068040	V2	None	TAN-1612	This study
			Aspfo1_0069798	V1	None	No data	This study
		*aculeatus*	Aacu16872_063282	V1	None	No data	This study
			Aacu16872_063333	V1	None	No data	This study
		*brasiliensis*	Aspbr1_0070836	V1	None	No data	This study
			Aspbr1_0071307	V2	None	TAN-1612	This study
		*clavatus*	EAW13612	V1	None	No data	This study
		*flavus*	EED53479	V1	None	No data	This study
			KJJ30826	V3	None	No data	This study
		*fumigatus*	EAL84397	V1	Endocrocin	Characterized	[47]
			EAL84875	V2	Neosartoricin, fumicycline	Characterized	[49,63]
			EAL89339	V1	Trypacidin	Characterized	[53]
			EDP47078	V1	Endocrocin	Characterized	[47]
			EDP47964	V2	None	Neosartoricin, fumicycline	This study
			EDP50840	V1	None	Trypacidin	This study
			KEY78897	V1	None	Endocrocin	This study
			KEY82310	V1	None	Other anthraquinone	This study
			KEY82351	V2	None	Neosartoricin, fumicycline	This study
		*glaucus*	Aspgl1_0045725	V1	None	No data	This study
		*kawachii*	GAA85937	V2	None	TAN-1612	This study
			GAA88581	V1	None	No data	This study
		*nidulans*	CBF70387	V2	Asperthecin	Characterized	[46]
			CBF79143	V3	Alternariol	Characterized	[54]
			CBF90097	V1	Monodictyphenone	Characterized	[51]
		*niger*	EHA20150	V1	None	No data	This study
			XP_001402309	V1	None	No product	This study
			AEN83889	V2	TAN-1612	Characterized	[64]
			CAK40778	V2	None	TAN-1612	This study
			CAK47960	V1	None	No data	This study
		*ochraceoroseus*	KKK15179	V1	None	Emodin	This study
			KKK17199	V1	None	No data	This study
		*oryzae*	BAE58990	V1	None	No data	This study
			BAE62229	V3	None	Alternariol	This study
			KDE80734	V3	None	Alternariol	This study
			KDE81226	V1	None	No data	This study
			XP_001820992	V1	None	No data	This study
		*parasiticus*	KJK64046	V3	None	No data	This study
		*rambellii*	KKK15908	V1	None	No data	This study
			KKK27047	V1	None	Other anthraquinone	This study
		*ruber*	EYE98259	V1	None	Endocrocin anthrone	This study
		*sydowii*	Aspsy1_0090693	V1	None	No data	This study
			Aspsy1_0144848	V1	None	No data	This study
			Aspsy1_0151845	V2	None	Asperthecin	This study
			Aspsy1_0157033	V2	None	Neosartoricin, fumicycline	This study
			Aspsy1_1049255	V1	None	No data	This study
		*terreus*	EAU31624	V1	Geodin	Characterized	[65,66]
			EAU37396	V1	None	No data	This study
			BAB88752	V1	None	No data	This study
		*tubingensis*	Asptu1_0059858	V2	None	No data	This study
			Asptu1_0123892	V2	None	TAN-1612	This study
		*ustus*	KIA75323	V1	None	Benzophenone	This study
			KIA75530	V2	None	Asperthecin	This study
			KIA75835	V2	None	Viridicatumtoxin-like naphthacenedione	This study
		*versicolor*	Aspve1_0089706	V2	None	Neosartoricin, fumicycline	This study
			Aspve1_0122449	V1	None	No data	This study
			Aspve1_0886277	V2	None	Asperthecin	This study
		*wentii*	Aspwe1_0034272	V1	None	No data	This study
		*zonatus*	Aspzo1_2112764	V1	None	No data	This study
	*Capronia*	*epimyces*	EXJ89638	V1	None	Other anthrone	This study
	*Cladophialophora*	*carrionii*	ETI19899	V1	None	Benzophenone	This study
		*yegresii*	EXJ61970	V1	None	Benzophenone	This study
	*Endocarpon*	*pusillum*	ERF75912	V1	None	No product	This study
	*Microsporum*	*gypseum*	EFR03594	V2	None	Neosartoricin, fumicycline	This study
	*Neosartorya*	*fischeri*	EAW20700	V2	Neosartoricin, fumicycline	Characterized	[49,63]
			EAW24682	V1	None	Benzophenone	This study
			EAW24697	V1	None	Grisandiene	This study
			EAW25724	V1	None	No data	This study
	*Penicillium*	*aethiopicum*	ADI24926	V2	Viridicatumtoxin	Characterized	[48]
			ADI24953	V3	Griseofulvin	Characterized	[48]
		*expansum*	KGO43750	V1	None	Benzophenone	This study
			KGO65172	V1	None	Emodin	This study
		*oxalicum*	EPS34273	V1	None	Benzophenone	This study
	*Talaromyces*	*cellulolyticu*	GAM33809	V1	None	No data	This study
			GAM37897	V1	None	Grisandiene	This study
			GAM40075	V3	None	No data	This study
			GAM42425	V1	None	No data	This study
			GAM43179	V1	None	No data	This study
		*islandicus*	CRG83532	V1	None	No data	This study
			CRG86674	V3	None	No data	This study
			CRG92129	V1	None	No data	This study
		*marneffei*	KFX46552	V1	None	Grisandiene	This study
			KFX52365	V1	None	Other anthrone	This study
			ADH01670	V1	None	No data	This study
			ADH01674	V1	None	No data	This study
		*stipitatus*	EED18910	V1	None	Emodin anthrone	This study
			EED18976	V1	None	Benzophenone	This study
	*Trichophyton*	*equinum*	EGE06343	V2	None	Neosartoricin, fumicycline	This study
		*interdigitale*	KDB28089	V2	None	Neosartoricin, fumicycline	This study
		*rubrum*	EZG11077	V2	None	Neosartoricin, fumicycline	This study
			KDB38561	V2	None	Neosartoricin, fumicycline	This study
		*soudanense*	EZF78756	V2	None	Neosartoricin, fumicycline	This study
		*tonsurans*	EGD99348	V2	None	Neosartoricin, fumicycline	This study
		*verrucosum*	EFE44835	V2	None	Neosartoricin, fumicycline	This study
Lecanoromycete	*Usnea*	*longissima*	AGI60157	V1	None	No data	This study
Leotiomycete	*Blumeria*	*graminis*	CCU75801	V3	None	No data	This study
			EPQ66189	V3	None	No data	This study
	*Botrytis*	*cinerea*	AAR90250	V3	None	No data	This study
			EMR83380	V3	None	No data	This study
			XP_001553397	V3	None	No data	This study
	*Oidiodendron*	*maius*	KIM92894	V1	None	Grisandiene	This study
			KIM93459	V2	None	No data	This study
			KIM96903	V2	None	No data	This study
			KIM99919	V3	None	No data	This study
	*Sclerotinia*	*borealis*	ESZ98980	V1	None	Benzophenone	This study
Sordariomycete	*Claviceps*	*purpurea*	CCE31584	V1	None	Benzophenone	This study
	*Colletotrichum*	*graminicola*	EFQ33703	V3	None	Alternariol	This study
		*sublineola*	KDN60962	V3	None	No data	This study
			KDN62802	V3	None	No product	This study
	*Diaporthe*	*ampelina*	KKY32371	V1	None	Benzophenone	This study
			KKY34489	V1	None	Endocrocin anthrone	This study
			KKY37364	V1	None	No product	This study
	*Eutypa*	*lata*	EMR67234	V1	None	Benzophenone	This study
	*Gaeumannomyces*	*graminis*	EJT69423	V3	None	Alternariol	This study
	*Grosmannia*	*clavigera*	EFX02748	V1	None	Benzophenone	This study
			EFX04268	V3	None	No product	This study
	*Metarhizium*	*acridum*	EFY89907	V2	None	Viridicatumtoxin-like naphthacenedione	This study
		*album*	KHN97625	V2	None	Viridicatumtoxin-like naphthacenedione	This study
		*anisopliae*	KID64953	V2	None	Viridicatumtoxin-like naphthacenedione	This study
			KID65337	V1	None	Grisandiene	This study
			KJK89454	V2	None	Viridicatumtoxin-like naphthacenedione	This study
			KJK95825	V1	None	Grisandiene	This study
		*brunneum*	KID65403	V1	None	Grisandiene	This study
			KID75074	V2	None	Viridicatumtoxin-like naphthacenedione	This study
		*guizhouense*	KID84413	V1	None	Grisandiene	This study
			KID87428	V2	None	Viridicatumtoxin-like naphthacenedione	This study
		*majus*	KID93731	V1	None	Grisandiene	This study
			KID97686	V2	None	Viridicatumtoxin-like naphthacenedione	This study
		*robertsii*	EXU97310	V2	None	Viridicatumtoxin-like naphthacenedione	This study
			EXV00352	V1	None	Grisandiene	This study
	*Myceliophthora*	*thermophila*	AEO58356	V1	None	Benzophenone	This study
	*Pestalotiopsis*	*fici*	AGO59040	V1	Pestheic acid	Characterized	[52]
			ETS76950	V1	Pestheic acid	Characterized	This study
	*Phaeomoniella*	*chlamydospora*	KKY14863	V3	None	No data	This study
	*Stachybotrys*	*chartarum*	KEY72288	V1	None	Benzophenone	This study
			KFA45466	V3	None	No data	This study
			KFA54249	V1	None	Benzophenone	This study
			KFA70942	V3	None	No data	This study
			KFA71118	V1	None	Benzophenone	This study
	*Thielavia*	*terrestris*	AEO66245	V1	None	Grisandiene	This study
	*Trichoderma*	*atroviride*	EHK46042	V1	None	No data	This study
		*harzianum*	KKO99957	V1	None	Other anthraquinone	This study
			KKP03797	V3	None	No data	This study
		*reesei*	ETR97748	V3	None	Alternariol	This study
		*virens*	EHK20655	V3	None	Alternariol	This study

## 3. Phylogenetics Directs Product Prediction

Directed by our phylogenetic analysis, we sought to predict the products of the uncharacterized members of group V by comparison to the characterized gene clusters. Coevolution of the genes in SM gene clusters allows that phylogenetic analysis of a constituent gene, or the protein it encodes, can be used to inform the search for clusters with interesting similarities or differences to known clusters. We applied the existing knowledge of the unique attributes of group V gene clusters to the clusters identified in our phylogenetic tree, and discuss examples from groups of gene clusters that are potentially of interest or that might be insignificantly different from known clusters. These results should aid in identifying interesting targets for future study and in avoiding duplication of efforts in the SM research community.

### 3.1. Group V1

**Figure 4:**
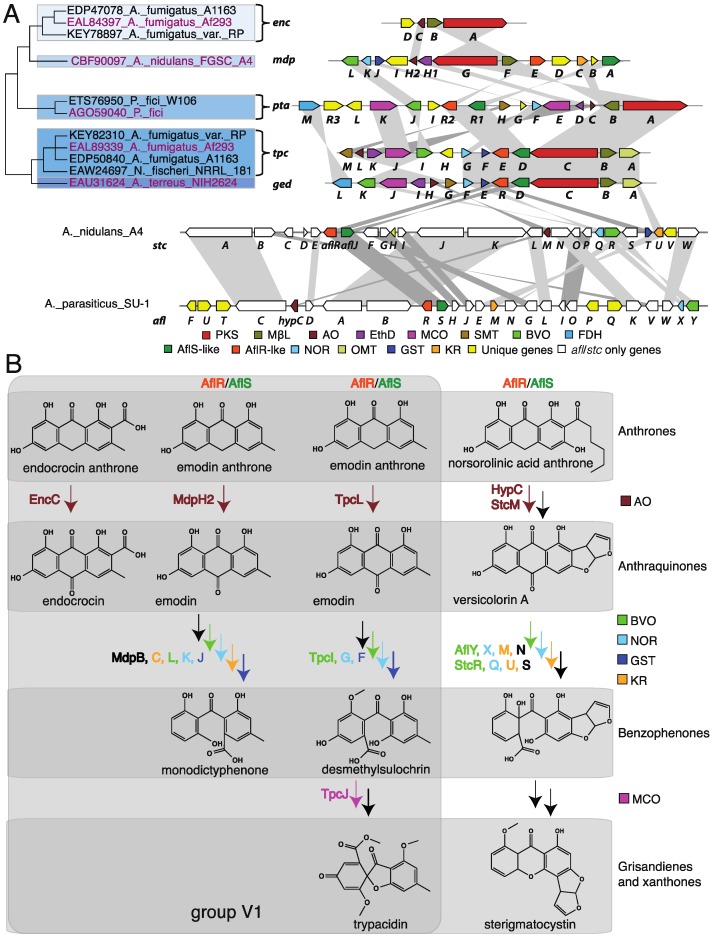
(**A**) A phylogenetic tree created from the group V maximum likelihood tree (Figure S2) showing just the relationships between the characterized group V1 PKSs. The gene cluster diagrams next to brackets depict the cluster corresponding to the PKS with its accession number highlighted in red, but all of the bracketed PKSs belong to clusters which are identical in terms of the presence and synteny of their group V-cluster homologs. Genes are represented as arrows with a color corresponding to their ortholog group and these are connected by shaded regions. Genes colored in yellow are unique among clusters shown here. Genes with no color in the *afl* and *stc* clusters do not have a homolog in the group V1 clusters shown; (**B**) A comparison of the analogous reactions catalyzed by the enzymes encoded by homologs of *afl* cluster genes in the endocrocin, monodictyphenone, trypacidin, and aflatoxin pathways. The reactions of the trypacidin pathway are representative of the geodin and pestheic acid biosynthetic pathways. Pathways of group V1 clusters are enclosed in a grey box. The enzymes catalyzing each reaction are shown to the left of the arrows and the color of the text and arrows matches Figure 4A. Arrows in black represent reactions not shown or reactions for which the enzymes, also labeled in black, are not homologous, except for AflN and StcS, which are homologous. PKS = Polyketide synthase, MβL = Metallo-β-lactamase-type thioesterase, AO = Anthrone oxidase, EthD = EthD domain-containing protein, a putative decarboxylase [51,53], MCO = multicopper oxidase, SMT = *S*-adenosylmethionine-dependent methyltransferase, BVO = Baeyer-Villiger oxidase, FDH = Flavin-dependent halogenase, AflS = Transcriptional co-regulator of the aflatoxin biosynthetic gene cluster [76], AflR = Transcriptional regulator of the aflatoxin biosynthetic gene cluster, NOR = NADH-dependent oxidoreductase, OMT = *O*-methyltransferase, GST = Glutathione *S*-transferase, KR = Ver-1-like ketoreductase [77,78].

Within the group V PKSs, a subset known as group V1 consists of octaketide synthases with C6–C11 cyclization. Five clusters from this subgroup have been characterized and their products determined. These include endocrocin, monodictyphenone, trypacidin, geodin, and pestheic acid, produced by *A. fumigatus*, *A. nidulans*, *A. fumigatus*, *A. terreus*, and *Pestalotiopsis fici*, respectively [47,51,52,53,66]. Many of the initial studies characterizing these clusters relied on earlier biochemical characterization of the geodin and aflatoxin biosynthetic pathways [50,65,67,68,69,70,71,72]. Group V1 is notable for the large number of aflatoxin homologs its clusters contain. Some clusters belonging to groups V2 and V3 have homologs of *aflL* (*vrtK*) and *alfO* (*gsfD*), but the characterized clusters in group V1 collectively contain homologs of as many as seven *afl* or *stc* (sterigmatocystin) cluster genes. These include homologs of *aflR*, *aflS*, *aflX*, *aflY*, *aflM*, *hypC*, and *stcT* (Figure 4A). The trypacidin and geodin clusters have previously been noted to contain partial aflatoxin clusters [73], but this is true of group V1 clusters in general. Manual analysis of our MultiGeneBLAST (MGB) [74] results additionally revealed a gene with significant similarity to versicolorin B-synthase (Vbs, AflK) associated with several uncharacterized clusters (Figure S2). Study of clusters containing *vbs* homologs might reveal an interesting role for this addition *afl* cluster homolog in group V. We speculate that the high number of *afl*/*stc* gene homologs reflects that group V1 clusters share a common ancestor with the *afl*/*stc* clusters.

All characterized group V1 clusters produce metabolites with an anthraquinone skeleton, such as endocrocin, emodin, and versicolorin A, by action of anthrone oxidases, e.g., HypC, StcM, EncC, MdpH2, and TpcL. Except for endocrocin which represents an end-product, a subset may then be processed into an open-ringed benzophenone structure, like monodictyphenone, by a Baeyer-Villiger oxidase (BVO), an NADH-dependent oxidoreductase (NOR), and potentially a glutathione *S*-transferase (GST). These reactions are similar to early steps in aflatoxin biosynthesis and this is reflected in the conservation of homologs for these key enzymes in group V1 clusters, though the sets of enzymes required between the aflatoxin/sterigmatocystin and group V1 cluster pathways are only partially overlapping (Figure 4B). Benzophenones may further be converted into closed-ring structures by spontaneous dehydration to xanthones, such as sterigmatocystin, or enzymatically to grisandienes, like trypacidin and geodin, by a multicopper oxidase (MCO), e.g., TpcJ. We leverage the knowledge of these conserved enzymes that catalyze the conversions between these chemical classes to make predictions about the products of related but uncharacterized clusters in this group (Table 1).

#### 3.1.1. Endocrocin-Like Clusters

The smallest characterized cluster from group V1, the four-gene endocrocin cluster was characterized as a virulence factor in *A. fumigatus* [47,75] and is also found in *Neosartorya fischeri*. A similar PKS is present in *A. terreus* (EAU37396), but appears to not be surrounded by any decorating genes, suggesting that the cluster might be a remnant of an endocrocin-like cluster. The endocrocin cluster encodes only two other enzymes in addition to the PKS (EncA) and MβL-TE (EncB) characteristic of group V clusters, an anthrone oxidase (EncC) and a 2-oxoglutarate-Fe(II)-type oxidoreductase (EncD). This minimal complement of decorating enzymes enables the production of the simple anthraquinone endocrocin. For our predictions of the products of uncharacterized group V1 clusters, the presence of genes encoding a PKS and an MβL-TE was considered sufficient to produce anthrones, with the additional presence of an anthrone oxidase-encoding gene required to predict an anthraquinone product (Table 1). Closely related PKSs in a series of other aspergilli are associated with homologs of *encB*, but no homologs of *encC* or *encD* were found in clusters that were available for analysis by MGB or AspGD. Similar to what was observed in *A. terreus*, it appears that the clusters in this clade may be remnants of endocrocin-like clusters (Figure S2). It is intriguing to speculate that cluster duplications from more complex clusters present in group V1 (see below) and subsequent deterioration led to this sub-group. Recent evidence for chemical redundancy between endocrocin and intermediates from the trypacidin cluster in *A. fumigatus* could explain the potential decay of the endocrocin and endocrocin-like clusters [53].

#### 3.1.2. Monodictyphenone-Like Clusters

The monodictyphenone-producing cluster in *A. nidulans* consists of 12 genes, notably including homologs of the three genes required for endocrocin production in the *enc* cluster. These encode a PKS (MdpG), an MβL-TE (MdpF), and an anthrone oxidase, MdpH2. Though the latter is annotated as part of a larger gene, *mdpH*, studies of the trypacidin- and geodin-producing clusters [53,66] suggest that *mdpH* is actually two separate genes, herein referred to as *mdpH1* and *mdpH2*. The *mdp* cluster is capable of producing endocrocin, but only in the absence of *mdpH* [51], suggesting that the other half of this gene, *mdpH1*, encodes a decarboxylase. This cluster additionally produces prenylated xanthones with the activity of prenyltransferases encoded outside of the cluster itself [79]. Among the proteins encoded by characterized group V cluster genes, MdpB, MdpC, MdpD, and MdpI are unique to the *mdp* cluster. The presence of the corresponding genes can be used to differentiate *mdp*-like clusters from clusters more similar to the other members of group V1.

Although not products of a group V PKS, the polyketide mycotoxins aflatoxin and sterigmatocystin share decorating enzymes to all group V1 pathways. Biosynthesis of aflatoxin and sterigmatocystin involves the conversion of the anthraquinone precursor versicolorin A to the xanthone demethylsterigmatocystin through a benzophenone-like intermediate and involves the actions of a cytochrome P450 monooxygenase (P450), AflN/StcS [67,68,80], a ketoreductase, AflM/StcU [77], an NOR, AflX/StcQ [70,77], and a BVO, AflY/StcR [71]. In the biosynthesis of monodictyphenone and prenyl xanthones in *A. nidulans*, a similar ring-opening reaction involving the conversion of the anthraquinone chrysophanol to the benzophenone aldehyde arugosins was recently proposed to be carried out by a glutathione *S*-transferase (GST), MdpJ, an NOR, MdpK, and a BVO, MdpL [51,78]. The genes encoding these enzymes bear significant similarity to StcT, AflX, and AflY, respectively (Figure 4). No known role is proposed for StcT in sterigmatocystin biosynthesis and no homolog of *stcT* is present in the aflatoxin gene cluster. Interestingly, action of only the BVO MdpL followed by hydrolysis is sufficient to produce the benzophenone carboxylic acid monodictyphenone as a shunt product. The *mdp* cluster also contains an *aflM* homolog in *mdpC*, but MdpC has been speculated to catalyze the conversion of emodin to chrysophanol in combination with MdpB and not to be involved in the ring-opening step [78]. The biosyntheses of the related compounds trypacidin and geodin in *A. fumigatus* and *A. terreus*, respectively, involve ring-opening conversion of the anthraquinone questin to the benzophenone desmethylsulochrin speculated to be catalyzed by a BVO (TpcI/GedK) and potentially an NOR (TpcG/GedF) and GST (TpcF/GedE) [53,66,67,68,78]. In pestheic acid biosynthesis in *P. fici*, the ring-opening of the anthraquinone physcion to the benzophenone desmethylisosulochrin is similarly proposed to be mediated by a BVO, PtaJ, and an NOR, PtaF, but this cluster encodes no GST, suggesting that this enzymatic activity might not be required for this transition [52].

In summary, these anthraquinone ring-opening reactions to form benzophenones all involve BVOs, NORs, and potentially GSTs. This is a similar but distinct set of enzymes required for the analogous chemical reactions in the biosynthesis of aflatoxin and sterigmatocystin. The presence of genes encoding these conserved anthraquinone ring-opening enzymes, a BVO and a NOR, in addition to the basic enzymes required to produce an anthraquinone, a PKS and MβL-TE, was used as the criterion for prediction of benzophenones as the products of many uncharacterized group V1 clusters (Table 1). Taken together, it is remarkable that, despite the obvious differences between the aflatoxin/sterigmatocystin PKSs (belonging to group IV) and the PKSs present in group V, this subset of enzymatic genes catalyzing ring-opening reactions is shared. Similar to this set of enzymatic genes, homologs of the two regulatory proteins AflR/S are also conserved in group V1 (Figure 4). It is noteworthy that, unlike the ring-opening enzymes, genes encoding AflR/S homologs can be found in other group IV clusters, *i.e.*, the fusarubin cluster [81], and even in group III clusters, *i.e.*, the bikaverin cluster [82].

One cluster from *A. ustus*, speculated to produce monodictyphenone in a recent study [83] due to its close phylogenetic relationship with that cluster, also has an MCO similar to that of the trypacidin, geodin, and pestheic acid clusters (see below). This suggests that this cluster might produce a chemical structure more similar to these latter clusters than to monodictyphenone and thereby exemplifies the need for a close evaluation of the whole cluster architecture.

#### 3.1.3. Trypacidin, Geodin, and Pestheic Acid-Like Clusters

As noted above, the monodictyphenone cluster shares many similarities with the trypacidin and geodin biosynthetic pathways in *A. fumigatus* and *A. terreus*, respectively, and pestheic acid biosynthesis in *P. fici*. All of these clusters catalyze anthraquinone to benzophenone ring-opening reactions using a BVO and an NOR. In the biosyntheses of trypacidin and geodin, the transition from the open benzophenone to closed grisandiene is catalyzed by a MCO. Specifically, in trypacidin biosynthesis TpcJ converts monomethylsulochrin to trypacidin, and, in geodin biosynthesis, GedJ converts dihydrogeodin to geodin [48,84]. The presence of a gene encoding an MCO in addition to the enzymatic machinery required to produce a benzophenone, a PKS, MβL-TE, BVO, and NOR, was used as the criterion for prediction of grisandienes as the products of many uncharacterized group V1 clusters (Table 1).

**Figure 5:**
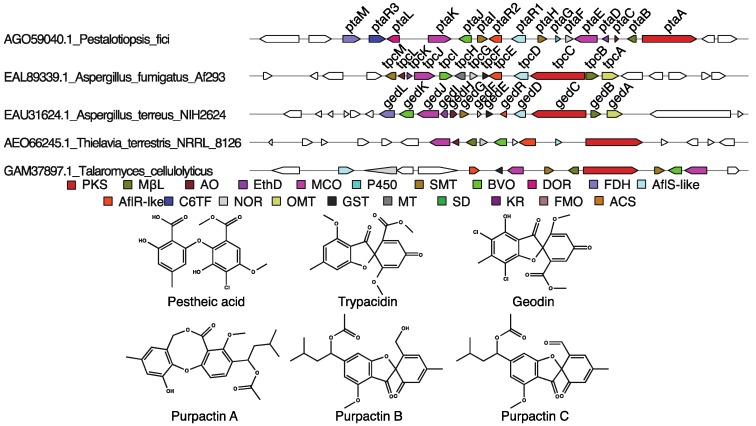
Examples of *tpc*-like clusters of interest. At top, the gene cluster diagrams are shown for the characterized *pta*, *tpc*, and *ged* clusters as well as two uncharacterized gene clusters from the group V1 chosen from the larger group V phylogenetic tree, Figure S2. Genes are represented as arrows with a color corresponding to the proteins they encode which are detailed in the color key below the cluster diagrams. Genes with no color were not identified as homologous to any group V1 cluster gene. The structures of the characterized products from this clade, pestheic acid, trypacidin, and geodin, are shown below the color key. Below these are three metabolites reported to be produced by *T. cellulolyticus* (now considered synonymous with *T. pinophilus*), purpactins A–C [87]. PKS = Polyketide synthase, MβL = Metallo-β-lactamase-type thioesterase, AO = Anthrone oxidase, EthD = EthD domain-containing protein, a putative decarboxylase [51,53], MCO = multicopper oxidase, P450 = cytochrome P450, SMT = *S*-adenosylmethionine-dependent methyltransferase, BVO = Baeyer-Villiger oxidase, DOR = Pyridine nucleotide-disulfide oxidoreductase, FDH = Flavin-dependent halogenase, AflS = Transcriptional co-regulator of the aflatoxin biosynthetic gene cluster [76], AflR = Transcriptional regulator of the aflatoxin biosynthetic gene cluster, C6TF = GAL4-like Zn(II)_2_Cys_6_-domain and fungal-specific transcription factor domain-containing protein, NOR = NADH-dependent oxidoreductase, OMT = *O*-methyltransferase, GST = Glutathione *S*-transferase, MT = Methyltransferase, SD = Scytalone dehydratase, KR = Ver-1-like ketoreductase [77,78], FMO = Flavin-dependent monooxygenase, ACS = Acyl-CoA synthase.

Comparison of the trypacidin, pestheic acid, and geodin clusters to clusters from other fungi allows us to speculate on products from undefined fungal clusters. A PKS (AEO66245) encoded by *Thielavia terrestris* is closely related to the geodin and trypacidin PKSs, and the cluster to which it belongs has homologs to eight of the thirteen genes in the trypacidin cluster (Figure 5). Importantly, these include genes encoding the enzymes required for the anthraquinone to benzophenone transition, a BVO and an NOR, as well as the benzophenone to grisandiene transition, which is catalyzed by a MCO in this subgroup of clusters. The presence of genes encoding these key enzymes suggests that this cluster might ultimately produce a grisandiene (trypacidin or geodin-like molecule). This fungus is a little-known species that can cause human infections [85]. Interestingly, trypacidin is a toxic spore metabolite produced by the opportunistic pathogen *A. fumigatus* [86], which may suggest that the metabolite produced by the *T. terrestris* cluster could play a role in pathogenicity.

Inspection of another of the PKSs closely related to the trypacidin, geodin, and pestheic acid PKSs, GAM37897.1 from *Talaromyces cellulolyticus* (now recognized as synonymous with *T. pinophilus*) (Figure 5), showed that the gene encoding this protein is part of a thirteen-gene cluster with homologs of nine of the thirteen genes in the trypacidin-producing gene cluster. This cluster also has genes encoding the key BVO and NOR enzymes and so likely produces a grisandiene, whether as an intermediate or an end-product. This species is known to produce many secondary metabolites including austin, mitorubrins, penicillides/purpactins/vermixocins, rubropunctatin, vermicellin, vermiculin, vermistatin and (3-*O*-methyl-, 3-*O*-methyl-5,6-epoxy-) funicones, MC-141, pestalacin A, stromemycin, dinapinone A1 and A2, and monoapinone A–E [87]. The structures of vermixocins and purpactins suggest they are products of this cluster. These compounds have grisandiene- and depsidone-like scaffolds, which are known or speculated to derive from the biosynthetic pathways of group V clusters such as geodin and pestheic acid [66,88]. Notably, these compounds appear to be prenylated despite the lack of a prenyltransferase identified in the cluster; however, the modification of SMs by prenyltransferases encoded outside of the gene cluster has been observed in monodictyphenone derivatives [79].

### 3.2. Group V2

**Figure 6:**
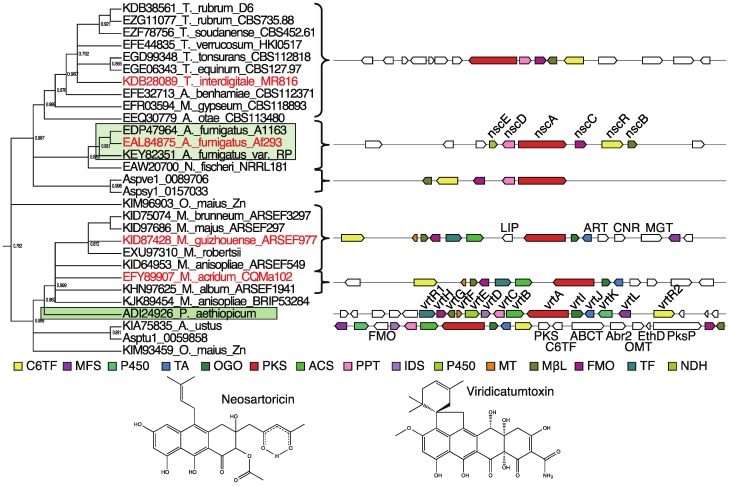
A clade of *nsc*-like clusters in *Trichophyton*, a clade of *vrt*-like clusters in *Metarhizium*, and other closely related clusters. An excerpt of the group V phylogenetic tree made with FastTree [60], Figure S2, containing the PKSs from the neosartoricin-producing cluster, NscA (EAL84875) [49], and the viridicatumtoxin-producing cluster, VrtA (ADI24926) [48], and groups of related uncharacterized PKSs, primarily in *Trichophyton* and *Metarhizium*, respectively, is shown at top left. The bootstrap values are presented next to their corresponding nodes. The green boxes indicate PKSs from the same species in which the characterized clusters were originally described. Next to the tree are the gene clusters corresponding to the PKSs that were identifiable through MultiGeneBLAST analysis. Gene cluster diagrams next to brackets depict the cluster corresponding to the PKS with its accession number highlighted in red, but all of the bracketed PKSs belong to clusters which are identical in terms of the presence and synteny of their *nsc*- or *vrt*-cluster homologs. Genes are represented as arrows with a color corresponding to the proteins they encode which are detailed in the color key below the tree and cluster diagrams. Genes with no color were not identified as homologous to any group V2 cluster gene. The products of the characterized examples from this clade, neosartoricin and viridicatumtoxin, are shown at bottom. C6TF = GAL4-like Zn(II)_2_Cys_6_-domain and fungal-specific transcription factor domain-containing protein, VrtR1-like, MFS = Major Facilitator Superfamily transporter, P450 = Cytochrome P450, TA = Threonine aldolase, OGO = 2-oxoglutarate-Fe(II)-type oxidoreductase, PKS = Polyketide synthase, ACS = Acetoacetyl-CoA synthase, PPT = Polycyclic prenyltransferase, IDS = Isoprenyl diphosphate synthase, MT = Methyltransferase, MβL = Metallo-β-lactamase-type thioesterase, FMO = Flavin-dependent monooxygenase, TF = GAL4-like Zn(II)_2_Cys_6_-domain and fungal-specific transcription factor domain-containing protein, VrtR2-like, NDH = NAD-dependent dehydratase, LIP = Secretory lipase, ART = Arrestin, CNR = Copper-containing nitrite reductase, MGT = Magnesium transporter, ABCT = ABC transporter, Abr2 = Conidial pigment laccase, OMT = *O*-methyltransferase, EthD = EthD domain-containing protein, putative decarboxylase, PksP = Conidial pigment polyketide synthase.

Another subgroup of group V is group V2, which includes nona- and decaketide synthases with C6–C11 cyclization. Characterized examples include asperthecin [46], viridicatumtoxin [48], TAN-1612 [64], and neosartoricin [49]. Enzymatic activities unique to this group include a fourth-ring cyclization facilitated by a flavin-monooxygenase (FMO) and MβL-TE combination. In contrast to the tricyclic (anthracene) backbones produced by most group V PKSs, two characterized examples in group V2 have the ability to generate tetracyclic (naphthacenedione) backbones, TAN-1612, and viridicatumtoxin. This ability depends on several factors including the ability to synthesize a long, *i.e.*, nona- or decaketide, backbone and the presence of both an MβL-TE with Claisen-cyclase activity and a unique FMO [64]. Though VrtA is only a nonaketide synthase, it accepts the very unusual malonamoyl-CoA starter unit produced by VrtB and VrtJ, and thus has a long enough chain for a fourth cyclization [48]. Phylogenetic analysis of polycyclic prenyltransferases (PPTs) associated with these clusters was previously used to identify a group of clusters with this triad of a unique PKS, MβL-TE, and FMO in dermatophytic fungi [89] (Figure 6).

#### 3.2.1. Asperthecin-Like Clusters

Asperthecin, associated with sexual spore color in *A. nidulans* (Palmer and Keller unpublished data), is produced from a three-gene cluster encoding the NR-PKS, the MβL-TE, and a FAD-dependent oxidoreductase [46]. As seen in Figure S2, the three close relatives *A. versicolor*, *A. sydowii*, and *A. ustus* contain the same cluster, which we hypothesize is also likely to be associated with ascospore color in these fungi. The presence of this minimal triad of genes encoding a PKS, MβL-TE, and FMO, as well as the close relationship of these PKSs to the characterized asperthecin-producing PKS, were the criteria used to predict asperthecin as a product of several uncharacterized clusters (Table 1).

#### 3.2.2. TAN-1612-Like Clusters

TAN-1612, identified in *A. niger* [64] and also present in *A. kawachii*, is produced by a five-gene cluster, and three of these genes are homologous to the *apt* cluster genes. In addition to the three proteins described above, this cluster is differentiated from the *apt* cluster by the presence of genes encoding a methyltransferase (MT) and a GAL4-like Zn(II)_2_Cys_6_-domain and fungal-specific transcription factor domain-containing protein (C6TF). It is tempting to speculate that TAN-1612 might be associated with ascospore pigmentation however the sexual stage of neither *A. niger* nor *A. kawachii* has been described yet for assessment of such a hypothesis. The presence of this set of five genes, as well as the close relationship of these PKSs to the characterized TAN-1612-producing PKS, were used as the criteria for predicting TAN-1612 as the product of several uncharacterized clusters (Table 1).

#### 3.2.3. Neosartoricin-Like Clusters

Neosartoricin is produced by a six-gene cluster in *A. fumigatus* and *N. fischeri* [49]. This cluster was also identified as producing the related fumicyclines [63]. The cluster’s PKS, NscA/FccA, produces a decaketide chain, the longest known of all PKSs so far described along with the TAN-1612-producing PKS, AdaA [90]. Five of the six genes in the *nsc*/*fcc* cluster are conserved in the dermatophytic genera, *Trichophyton*, *Arthroderma*, and *Microsporum* (Figure 6) some of which have previously been noted [89]. Notably, the gene for which there is no conserved homolog in these species, the NAD-dependent dehydratase (NDH)-encoding *nscE*/*fccE*, has no proposed role in neosartoricin or fumicycline biosynthesis in *A. fumigatus* [49,63]. Compared to the *nsc* cluster, these conserved clusters in the Arthrodermataceae appear to have two two-gene inversions, but are otherwise syntenically conserved. Four of these five genes are homologous to genes in the TAN-1612-producing cluster of *A. niger*. The neosartoricin-producing cluster is differentiated from TAN-1612-producing clusters by the presence of genes encoding a PPT and an NDH and the absence of a homolog of the MT-encoding gene from the TAN-1612-producing cluster. The presence of the unique set of five genes of the *nsc* cluster, excluding the NDH-encoding *nscE*, as well as the close relationship of these PKSs to the characterized neosartoricin-producing PKS, were used as the criteria for predicting neosartoricin and fumicycline as the product of a group of uncharacterized clusters (Table 1). Fumicyclines are induced in the presence of *Streptomyces rapamycinicus* and neosartoricin has demonstrated immunosuppressive activity [49,63]. This potentially suggests an important role for this cluster in virulence of dermatophytes.

#### 3.2.4. Viridicatumtoxin-Like Clusters

Viridicatumtoxin is a tetracyclic mycotoxin produced by *Penicillium* species. The 14-gene *vrt* cluster contains homologs of the 5 conserved *nsc* genes mentioned above. We have identified a group of *Metarhizium* species that have gene clusters with homologs of ten of the *vrt*-cluster genes (Figure 6). *Metarhizium* species are entomopathogenic fungi in the Clavicipitaceae family. These *Metarhizium* clusters contain two regions of conserved synteny with four and five gene regions of the *vrt* cluster and are predicted to yield a tetracyclic polyketide. Interestingly, in *A. ustus* a *vrt*-like cluster contains homologs of *abr2* and *pksP* of the conidial pigment biosynthetic gene cluster, suggesting that this may be one large cluster or two interwoven clusters perhaps similar to that of the intermingled fumagillin/pseurotin supercluster in *A. fumigatus* [91]. Several of these species have been noted to have *vrt*-like clusters in recent studies [83,92]. The presence of the minimal triad of genes encoding a PKS, MβL-TE, and FMO, multiple other homologs of genes unique to the vrt cluster amongst group V cluster genes, as well as the close relationship of these PKSs to the characterized viridicatumtoxin-producing PKS, were used as the criteria for prediction of “viridicatumtoxin-like naphthacenedione” as the product of a group of uncharacterized clusters (Table 1).

### 3.3. Group V3: Griseofulvin and Various Alternariol-Like Pathways

**Figure 7:**
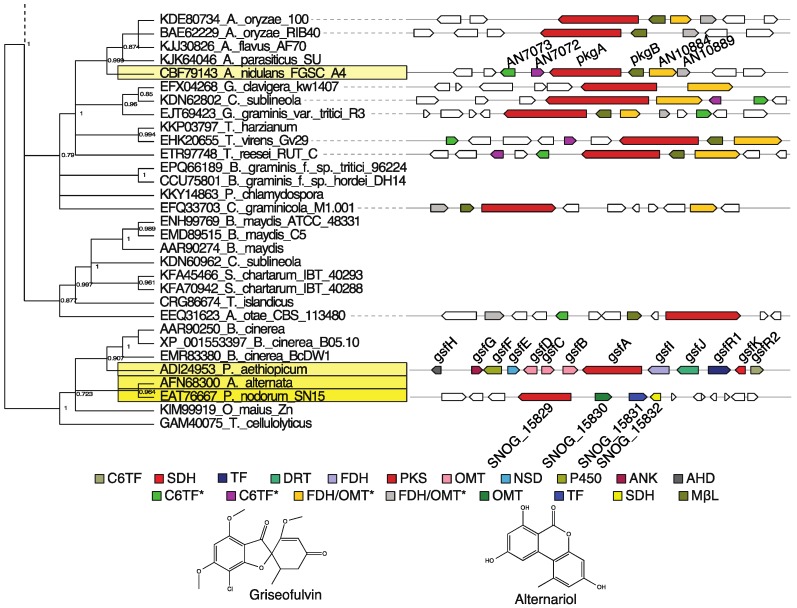
A clade of *gsf*-, *pkg*-, PskI-, and SNOG_15820-like clusters. An excerpt of the group V phylogenetic tree made with FastTree [60], Figure S2, containing the PKSs from the griseofulvin-producing cluster, GsfA (ADI24953) [48], three alternariol-producing clusters [54,61,62], and a group of related uncharacterized PKSs, is shown at top left. The bootstrap values are presented next to their corresponding nodes. The yellow boxes indicate PKSs from the same species in which the characterized clusters were originally described. Next to the tree are the gene clusters corresponding to the PKSs that were identifiable through MultiGeneBLAST analysis. Genes are represented as arrows with a color corresponding to the proteins they encode which are detailed in the color key below the tree and cluster diagrams. Asterisks signify potential gene truncation due to misannotation. Genes with no color were not identified as homologous to any group V3 cluster gene. The products of the characterized examples from this clade, griseofulvin and alternariol, are shown at bottom. C6TF = GAL4-like Zn(II)_2_Cys_6_-domain and fungal-specific transcription factor domain-containing protein, GsfR2-like, SDH = Short chain dehydrogenase, TF = GAL4-like Zn(II)_2_Cys_6_-domain and fungal-specific transcription factor domain-containing protein, GsfR1-like, DRT = Drug resistance transporter, EmrB subfamily, FDH = Flavin-dependent halogenase, PKS = Polyketide synthase, OMT = *O*-methyltransferase, NSD = Nucleoside-diphosphate-sugar dehydratase, P450 = Cytochrome P450, ANK = Ankyrin repeat-containing protein, AHD = YcaC-related amidohydrolase, FDH/OMT = Flavin-dependent halogenase and *O*-methyltransferase bifunctional protein, MβL = Metallo-β-lactamase-type thioesterase. * Asterisks signify potential gene truncation due to misannotation.

This group V subgroup includes heptaketide synthases catalyzing an unusual C1–C6 or C2–C7 first cyclization followed by a C8–C13 second cyclization. Characterized examples include alternariol and griseofulvin. Alternariol is an important mycotoxin produced by members of *Alternaria*, *Aspergillus*, and *Phaeosphaeria* [54,61,62]. This metabolite is a fairly common crop contaminant with carcinogenic, phytotoxic, and antifungal activity. Despite its importance, genetic studies characterizing the biosynthesis of alternariol were only recently undertaken. To date, three gene clusters have been implicated in the synthesis of alternariol, one in *Alternaria alternata*, one in *A. nidulans*, and one in *Phaeosphaeria nodorum* (syn. *Parastagonospora nodorum*) (Figure 7). Initially, PksJ (AFN68301) was identified in *A. alternata* using siRNA and gene deletion approaches as the PKS primarily responsible for alternariol production. However, two other PKSs, PksH (AFN68299) and PksI (AFN68300), were shown to be affected by the knockdown of *pksJ* expression. Notably, no MβL-TE was identified adjacent to PksJ in this study [61]. In *A. nidulans*, promoter replacement experiments were used to show that PkgA (CBF79143) and PkgB produce alternariol and coumarins [54]. Most recently, in *P. nodorum*, SNOG_15829 (EAT76667) was also found to produce alternariol. The cluster associated with this NR-PKS includes a gene encoding an MβL-TE (SNOG_15826), but it bears little similarity to the other MBL-TEs of group V clusters [62], which could be due to poor sequence quality. Interestingly, the NR-PKS EAT76667 is most similar to PksI from *A. alternata*, suggesting that PksI, and not PksJ or PksH, is the alternariol-producing PKS in *A. alternata*. These clusters were not characterized further than the identification of a PKS and an MβL-TE; the additional genes analyzed by MGB as part of the PkgA (CBF79143) and SNOG_15829 (EAT76667) clusters were included based only on their reported co-regulation with the PKS- and MβL-TE-encoding genes [62,93]. Because of these limitations in the studies characterizing alternariol-producing gene clusters, our product predictions for group V3 PKSs are based only on the presence or absence of genes encoding a PKS and MβL-TE and the closeness of the relationships of these PKSs with the characterized group V3 PKSs (Table 1).

Griseofulvin, produced by *Penicillium* species, is an antifungal drug widely used against dermatophytic infections [94,95,96]. Despite its resemblance to other grisandienes like trypacidin and geodin, the griseofulvin biosynthetic pathway [48] is quite unique and constitutes an interesting example of convergent evolution at the biochemical level. The NR-PKS responsible for griseofulvin production, GsfA (ADI24953), generates a benzophenone directly and by a different cyclization register than that of the group V1 PKSs MdpG, TpcC, GedC, and PtaA, *i.e.*, C1–C6 as opposed to C6–C11 for group V1 PKSs or even C4–C9 for the aflatoxin-producing group IV PKS, AflC [84]. Thus, whereas the biosynthetic pathways of group V1 clusters proceed through an anthraquinone intermediate to a benzophenone intermediate by action of a BVO, GsfA synthesizes a benzophenone as its initially released product. This is made possible by the unusual C1–C6 and C8–C13 connections it catalyzes. This latter connection is also observed in the biosynthesis of alternariol, but the two differ in their initial cyclization, *i.e.*, C1–C6 for griseofulvin and C2–C7 for alternariol [62]. Further, the benzophenone to grisandiene transition in griseofulvin biosynthesis is catalyzed by a cytochrome P450 as opposed to a MCO in the cases of trypacidin, geodin, and pestheic acid [97]. Curiously, the release mechanism of GsfA has yet to be elucidated, as it lacks the MβL-TE that is characteristic of group V. We speculate that the unique cyclization catalyzed by these PKSs might obviate the MβL-TE activity and explain the lack of an MβL-TE in the *gsf* cluster and the dissimilarity of the MβL-TE of the *P. nodorum* alternariol-producing cluster. It has been noted that the close relationship of the griseofulvin- and alternariol-producing PKSs is likely not coincidental and that unusual modes of cyclization may be unique to this clade [57].

## 4. Uncharacterized Clusters in Symbionts and Pathogens

As expected, considering the number of sequenced *Aspergillus* species, many of the NR-PKSs identified from this study are from *Aspergillus* species. However, of the non-*Aspergillus* genera and species, many are symbiotic, including pathogenic fungi associated with specific hosts. These findings support reviews of pathogenic fungi which highlight the potential role of secondary metabolites in virulence [1]. Below we touch on this emerging theme from our analysis.

### 4.1. Plant Pathogens

Several plant pathogenic fungi are present in two main groupings in Figure S2. Notably, the subclade to which the griseofulvin and alternariol PKSs belong contains many plant pathogenic species (Figure 7), including the pine pathogen *Grosmannia clavigera*, the sorghum pathogen *Colletotrichum sublineola*, the wheat pathogen *Gaeumannomyces graminis*, the maize pathogen *Bipolaris maydis*, the grape pathogen *Botrytis cinerea*, and two grass powdery mildew species from the genus *Blumeria*. This latter finding is especially intriguing as the genomes of obligate biotrophs such as powdery mildews contain few secondary metabolite genes [98,99]. As alternariol produced by related clusters from *Alternaria*, *Aspergillus*, and *Phaeosphaeria* spp. is known to be an important phytotoxin, it is possible these NR-PKSs produce a metabolite important in the fungal/plant host interaction.

Several plant pathogenic fungi also are present in the subclades producing trypacidin, geodin, pestheic acid, and monodictyphenone. The genus *Bipolaris* and allied genera *Setophaeria* and *Pyrenophora*—all grass pathogens—are particularly well represented in this clade. Another grass pathogen, *Claviceps purpurea*, is also present in this subclade, although it is taxonomically distant from the *Bipolaris* species and is best known for its suite of alkaloid-producing clusters [100]. It would be interesting to see if these clusters produce a metabolite specialized to interactions with grass hosts.

### 4.2. Mycorrhizal Fungi

*Oidiodendron maius* belongs to the rare group of fungi establishing mycorrhizal relationships with the plant family Ericaceae (heather) [101,102]. Most unusually, this species contains four NR-PKSs associated with every group V subgroup. Poor annotation of the genome prevented full assessment of the associated clusters; hopefully future sequencing efforts will provide data for accurate cluster predictions.

### 4.3. Fungal Pathogens

The mycoparasitic genus *Trichoderma* is represented twice in our analysis, once where they contain a gene cluster quite similar to the *pkg* cluster (PKS = CBF79143) and one similar to the pestheic acid cluster of group V1. Considering the mycoparasitic lifestyle of these fungi, the presence of these clusters raises the question whether their respective products could play a role in mycoparasitism.

### 4.4. Animal Pathogens

Several animal pathogens, from insect to human, contain NR-PKSs also found throughout group V. *Entomopathogenic fungi*. The genus *Metarhizium*, along with *Beauveria bassiana* not represented in Group V, is well known for its potential in biological control of various insects [103]. A putative group V1 cluster was identified in *B. bassiana* including EJP67854, but it did not include an annotated PKS and so was excluded from further analysis. It is unclear whether this is related to any of the several NR-PKSs that have previously been noted in *B. bassiana* [104]. As mentioned earlier, several species in *Metarhizium* contain clusters with significant similarity to the *Penicillium* viridicatumtoxin cluster (Figure 6). The *Metarhizium* genus is also well represented in group V1, suggesting the ability to produce a metabolite similar to these compounds. Toxicity is associated with all of these metabolites and may afford virulence properties to these insect pathogens.

*Dermatophytes*. Several dermatophytic genera, *Trichophyton*, *Arthoderma*, and *Microsporum* contain a neosartoricin-like gene cluster. This compound exhibits antiproliferative activity which may be suggestive of an immunosuppressive role in human infection by dermatophytic fungi [49]. The causal agent of a devastating bat disease in North America known as white nose syndrome, *Pseudogymnoascus destructans*, is a dermatophyte of bats [105]. This species and other members of the genus are found in two sections of group V, both grouping to the trypacidin, geodin, pestheic acid, and monodictyphenone clades. Metabolites produced by these clusters could possibly play a role in virulence of *P. destructans*.

## 5. Experimental Section

To retrieve the amino acid sequences of NR-PKSs in our initial search, the monodictyphenone PKS, MdpG [51], was analyzed with the NCBI’s BLASTP [106] against fungi (taxid: 4751) with max target sequences increased to 1000 and other parameters set to defaults. A similar search was executed on AspGD (aspgd.org) and 25 non-duplicate PKSs were identified from the top 50 hits and added to the list retrieved from the NCBI. The human fatty acid synthase, FASN, was added as a marker for the outgroup, which also included 70 HR-PKSs and hybrid PKS/NRPSs. The KS domains for these 908 PKSs were retrieved using the NCBI’s Conserved Domain Database (CDD) utility [107,108,109] and aligned using MAFFT [110] with default parameters. Alignment columns with greater than 40% gaps were removed using TrimAl [111], and the sequences were realigned. A maximum likelihood phylogenetic tree was constructed using FastTree [60] in Geneious 8.1.5 [112] with 1000 bootstrap replicates and otherwise default parameters. Nodes with bootstrap support values of less than 70% were collapsed to polytomy in Figure 6, Figure 7, and Figure S2. This tree was modified for presentation using FigTree [113] (Figure 2, Figure S1). To extract the sequences for the group V KS domains, a neighbor-joining tree was constructed in Geneious with default parameters, and the sequences for the smallest monophyletic group containing the characterized group V PKSs were selected and exported as a sub-alignment. These 188 sequences were realigned with MAFFT and used to construct a maximum likelihood phylogenetic tree using FastTree as above. This tree was modified for presentation using FigTree [113] (Figure 3, Figure S2). Excerpts of this tree were used to create Figure 4, Figure 5, Figure 6 and Figure 7.

MultiGeneBLAST [74] (MGB) was used to facilitate the analysis of uncharacterized clusters associated with the PKSs identified by the methods described above. MGB architecture searches were executed with the synteny conservation weight set to 0, the percent identity threshold set to 25%, the maximum intergenic distance threshold set to 25 kb, and otherwise default parameters. MGB searches were carried out with multifasta files containing one representative of each group of orthologous genes from a given subclade of group V, e.g., the group V1 search used a file containing one PKS, one MβL-TE, *etc.* (Tables S1–S3). If multiple genes in a single cluster encoded similar types of proteins, e.g., C6TFs, TFs, P450s, or MTs, they were considered as separate and included in the multifasta file for that subgroup (*i.e.*, V1, V2, or V3) despite their potential redundancy. This was done to identify potential patterns in the distribution of orthologs across other clusters, *i.e.*, if a given ortholog was more similar to one or the other of the potentially redundant genes. Alternatively, if the potentially redundant genes were not differentiable with our MGB parameters they and all their orthologs were considered to encode the same type of protein and represented as such in the cluster diagrams and corresponding color keys. The database available online (http://multigeneblast.sourceforge.net/index.html) containing all GenBank entries, updated 1/2015, was used to obtain the majority of the cluster diagrams (Figure S2, Figure 4, Figure 5, Figure 6 and Figure 7). Others were obtained from a custom fungal database generated by downloading 578 annotated fungal genomes from NCBI using a custom python script (https://github.com/nextgenusfs/NR-PKS_ms/get_ncbi_genomes.py). These genomes were then incorporated into a MGB database using the `makedb` program from the command line distribution of MGB. Still others, such as the *T. cellulolyticus* (GAM37897) and *A. terreus* (EAU31624) clusters (Figure 5, Figure S2), were manually created or modified (to reflect re-annotation of ATEG_08457 [66]), respectively. The protein descriptors in the keys or adjacent to the cluster diagrams (Figure 4, Figure 5, Figure 6 and Figure 7, Figure S2) were derived from the function or conserved domain of the protein according to published studies describing group V SM gene clusters [46,47,48,49,51,52,53,54,61,62,63,64,66,78] or individual BLAST searches.

## 6. Conclusions

The potential to synthesize polyketides is widespread in the fungal taxa ascomycetes and basidiomycetes, but examination of biochemical PKS classes indicates taxonomic specificities [57]. Illustrating this point, the TE-less NR-PKSs described in this work and others [54,57], group V, are notably absent in certain mycotoxigenic genera (e.g., *Fusarium*) and basidiomycetes but, surprisingly, found in genera not noted for secondary metabolism (e.g., *Oidiodendron* and *Blumeria*) (Figure 5, Figure 6 and Figure 7, Figure S2). Many of the described products derived from these NR-PKSs exhibit toxic activities. For example, questin, trypacidin, and endocrocin have been assessed for their impact on virulence as they are produced by the human pathogen *A. fumigatus*. Trypacidin and endocrocin in particular have been shown to exhibit toxic and neutrophil inhibitory properties, respectively, in pathogenicity studies [75,86]. The other *A. fumigatus* group V metabolite, neosartoricin, exhibits T-cell antiproliferative activity, which may be suggestive of an immunosuppressive role in human infection [49]. Furthermore, griseofulvin is a potent antifungal [94], viridicatumtoxin is a mycotoxin [114,115], and alternariol is known for its phytotoxic properties [116,117]. Thus, it is not improbable that the products of the NR-PKS clusters identified in the pathogenic and symbiotic fungi in this study (Figure S2) could impact fungal/host interactions, as virulence factors or signaling molecules. However, it is also possible that such roles could be coincidental or in addition to other functions in fungal biology.

As predicted by their structure, it is likely all are UV absorbing pigmented molecules observed in the visible yellow-orange spectrum. Possibly one significant role of group V PKSs may lie in protection or development of spores. Asexual spores are common, air-dispersed spores essential for propagation in the kingdom Fungi and must be equipped with defenses against abiotic stresses, such as UV radiation, oxidative stress, and desiccation. Several studies have shown that loss of pigmentation of spores leads to reduced viability and/or virulence in pathogenic fungi [118,119,120,121]. Sexual spores are the product of meiosis and essential for genetic recombination and species diversity and, in several species, are also airborne spores that would be exposed to UV radiation similarly to asexual spores [122,123]. Both trypacidin and endocrocin are produced in the asexual spore [75,86] and our studies (Palmer and Keller, unpublished data)—supported by an earlier investigation [124]—suggest that asperthecin is the red pigment characterizing the color of *A. nidulans* sexual ascospores. Assessment of spore viability of NR-PKS fungal mutants under UV conditions might shed light on a conserved role of these molecules to protect from specific abiotic stresses.

In this study we have examined all of the group V NR-PKSs available from NCBI and all of the corresponding gene clusters that were readily available using MultiGeneBLAST. The relatively high number of studies characterizing SM gene clusters from group V [46,47,48,49,51,52,53,54,61,62,63,64,66,78], has enabled us to predict the products of uncharacterized clusters in this group (Table 1). For group V1 this is largely based on the presence or absence of genes encoding key enzymes known to catalyze particular reactions, e.g., anthraquinone ring-opening by BVOs and NORs or benzophenone ring-closure by MCOs. For groups V2 and V3 these predictions are based only on the presence of homologs of a majority of the genes in the most closely related characterized group V cluster, e.g., neosartoricin is predicted to be produced from clusters containing homologs of five of the six *nsc* cluster genes. This study demonstrates that, by comparison to characterized examples in a given group of NR-PKSs, predictions can be made from phylogenetic analysis and used to help choose SM gene clusters to characterize. For example, future research efforts might be better spent studying SM gene clusters of interest highlighted by this study and not on those that are highly similar to characterized clusters and therefore likely to produce a known compound. Application of these methods to other groups of NR-PKSs might provide a similarly beneficial perspective.

## Supplementary Materials

Supplementary materials can be accessed at: http://www.mdpi.com/2072-6651/7/9/3572/s1.
