# Supplementary material for: Evolution of Chemical Diversity in a Group of Non-Reduced Polyketide Gene Clusters: Using Phylogenetics to Inform the Search for Novel Fungal Natural Products
Source: Toxins (Basel). 2015 Sep 10;7(9):3572–607. doi: 10.3390/toxins7093572 (PMC4591646; doi:10.3390/toxins7093572)
Supplement: Supplementary file 1 [file toxins-07-03572-s001.pdf]

Supplementary Information

Table S1. The composite multifasta file used for the group V1 MultiGeneBLAST search. This list contains one member of each group of orthologous genes in all group V1 clusters.

>gi|514825998|gb|AGO59040.1| PtaA (*Pestalotiopsis fici*)

MSDNSGSGTSPWGSLNTPVGPPKVTLAYFSNEFPPDDLNFIVRKLFDRTSKGPFCSIDGVLLCAIQFAN
LIGHYETTDHLFPFGSSIASVAGLGIGLVAAAAVSVTPSLADLPVAGAEAVRIAFRLGVLVDGVSQNL
QPRDRSTTGTPDSWAYVIPDVSPEVVQKELDEIHSREKTPIPSKIFVSALSRTSVTISGPPARLRSLFRLS
DFFRDRKFVALPVYGGLCHAGHIYEQRHVQEVVEKSVLDETHVRYSPSVRLFSTSTGKPFLSTSVTN
LFEQVVGEILTQKIQWDKVVKGVLERIQELSATEVEVLVFRDSLPVHELVKALKSADSGLQTTTEDL
LQWLHQSRERLQGPRGSLQSKIAIVGMSCRMPSGATDTEKFWELLEKGLDVHRKIPADRFDVETHH
DPTGKRVNTSITPYGCFIDEPGLFDAGFFNMSPREAQQTDPMQRLALVTAYEALERAGYVANRTSAT
NLHRIGTFYGQASDDYREVNTAQEISTYFIPGGCRAFGPGRINYFFKFSGPSYSIDTACSSSLATIQAAC
TSLWNGDTDTVVAGGMNVLTNSDAFAGLGNGHFLSKTPNACKTWDCEADGYCRADGIGSIVMKR
LEDAEADNDNILGVILGAGTNHSADAISITHPHAPSQAFLYRQILRDAALDPFDVSFVEMHGTGTQAG
DSEEMQSVTEVFAPIANKRRTSKQPLHIGAVKSNVGHGEAVAGVTALIKVLLMFQKEAIPPHAGIKN
SINPGFPKDLDKRNINIPYQKTAWPRSTDRKRIAVVNNFSAAGGNTTIAIEEGPLRQTIGHDPRTTHLIP
ISAKSKVSLKGNIQRLIDYLEVSPDVSLADLSYSLTARRYHHSHRVAITTSDVAHLKKQLRSQLDSAD
SHKPIVAAAGPPPVAFAFTGQGASYGTMDLELYHESKYFRDQILQLDSFAQGQGFPSFVPAIDGSFPK
EHTHRPVVTQLALLCTEIALAKYWASLGVKPDVVIGHSLGEYAALHVAGVLSASDAIFLVGQRALM
LEKKCQAGSHKMLAVRASLAQVQEAAGELPYEVACINGQKDTVLSAAKDDIDKLASVLESAGYKC
FSLDVAFAFHSAQTDPILDDFESVSRTGVLFQAPNLPVISPLLGKVVFNDKTINANYVRRATRESVDF
LSALEAAQKISIIDESTTWIEIGPHPVCMGFIRSAVPSIKVASPSIRRGENNWQTLVQTLGALHLAGIPV
DWNEYHRPFEQALRLLDLPTYSWNDKTYWIQYNGDWALTKGNTFYDAEKAAKAPRVGGDLPPSPI
STSTVHRVIGETFDGTAGTVDIQSDLMQQDFHDAAYGHKMNNCGVVTSSIHADIVYTIGRYLHTKLK
PGVKDIHMNISNLEVVKGLVAQKNRDVPQLIQVSISTEDISSGTAQVTWFNVLPDGGLDEPFATATLF
YGKANDWLQSWIPTTHLVLGRVHELERLAEQGVANRFSRNMAYGLFARNLVDYADKYRGMQSVV
LHGLEAFADVELTKEKGGTWTVPPFFIDSVAHLAGFIMNVSDAVDTANNFCVTPGWESMRFARPLL
AGARYRSYVKMIPTEEDAGVFLGDVYIFQDNKIIGQVRGIKFRRYPRLLLDRFFSAPDAAKHGGKHA
PAVKAAIPPALEKKSAVVVAQVPVVDKPPPTKENAVAAPAAKSPEPVAAAAVNEDSITVKAMALVA
AEAALDVSELEDDVQFANIGVDSLMSLVIAEKFRETLGVTISGSLFLEYPAVGDLRAWLLEYYG

>gi|514825999|gb|AGO59041.1| PtaB (*Pestalotiopsis fici*)

MVGKGGYRQINKALNICAFEDYLDGQQKSLPPLNDVEQISPNVLRVLGQNPGKFTLQGTNTYIIGTG
EKRLLIDTGQGIPEWADLISSTLANSSIRLSAVLLSHWHGDHTGGVPDLLRLYPHLSDSIYKHSPSKGQ
QPIEDGQVFEVEGATVRAVHAPGHSHDHMCFVIEEENAMFTGDNVLGHGTAAVELLSTWMATLRL
MQSHNCGRGYPAHGEVIPNLNAKISGELASKERRERQVLQHLNRIRKEEQGGKGSATVQRLVVEMY
GDTDQQMREQALEPFIDEVLRKLAEDEKVAFQLRAGEKTWFAIALE

>gi|514826001|gb|AGO59043.1| PtaC (*Pestalotiopsis fici*)

MMGLPLMAVPMLLDTGADPVYLARQWARMYYYGVRTMPPLAITTFILYVWTIIRRRSQHQAWYIL
AVAAVVTMGMIPFTWYVLAPTNNALFRLAEGPEAASGTTAGSLEEVTELLVRWNKLHIARSLFPLT
GVVIALSDAM

>gi|514826002|gb|AGO59044.1| PtaD (*Pestalotiopsis fici*)

MAEMSKNPASTLKPSRYLCLTICGYRKPGMSEEDYRYHMTQVSAPMTKDLMAKYGVKRWTMNY
MIRRWPSCPTLTASAKLCLRTLEDYKRMKQDPWYKEHLIGDHEKFADTKRSKMTIGWIEEWISDGK
PVDGLEFKS

Table S1. *Cont.*

**>gi|514826000|gb|AGO59042.1| PtaE (*Pestalotiopsis fici*)**
MVIRTPFSIRLYESRPGMMFAMFQSILFLAFYGRPVFGSAAARDYACVNTAESRDCWKDGF<br>NIETDYYGKEEAPEGKLVEESTVKNNFTENYNGTAVHWHGIRQKETNWLDGVPGVTQCPI<br>TPGDSQVYEFRVTQYGTSWYHSHYSLQLMELMLMQSQGPIVIHGPSSANWDVDLGPWLLS<br>DWYHDDAFALDHVGITTNRAAIPKSSLINGKGYYECDPTNDAKCTGTRDYYEVVLKQGTK<br>YKFGIINTSTILTYTFWIDGHNFTIIAIDFVPIEPLTVDTLNVGIGQRYEIIIETNPDFDDDSSFW<br>MHAQYCFINQTDIVDDKVGIVRYESAGSSDPPYINKSDYHLNFGCADPKPESLVPILKQQVG<br>AQANPLAAEDYFRVGLGNFTWPDATNSTGSVFLWFLQKLPLYVNWSEPSVKKLTIDETADF<br>PPNSRPIELDYETGQWVYFVIESDWDPAGAVDQYGQEIRVEPSVHPFHLHGHDFLILAQGLG<br>KFTSDIQPNLDNPPRRDTVDVEPLGYVWIAFQIDNPGAWLFHCHIAFHSSDGIAIQFLEQPSK<br>LKPIMEEAGVLGDFADRCNKWDDWYQAVNIPHNATQADSGV
**>gi|514825993|gb|AGO59035.1| PtaF (*Pestalotiopsis fici*)**
MSRYAILGSTGNCGTALIENVLDSSMTEVHAFCRNQEKLERLVPRVISDARVKVFVGGIGDT<br>ETLAACLHGCNAVFLCITTNDNVPGCRVAQDTALGVVKVLERSRADGFLPMPKLVLLSSAT<br>IDDVLSRNTPWVLRSILLKSASHVYEDLRKTEILLRAEQDWLTTIFIKPGALSVDIQRGHALS<br>LTDEDSPVSYLDLAAAMIEAVNDPQGRYDMRNVGVVNTHGRANFPSGTPLCIAVGLLSHF<br>APFLHPYLPSGTGPR
**>gi|514826003|gb|AGO59045.1| PtaG (*Pestalotiopsis fici*)**
MASTGSAQKETLNKFISGWKNANAEEMLAVASDDYTQQTLPFSLGHDVRPKQVAEVMLP<br>KLYSILENYELKIHQVVHDVENQKAAVYAISKADTPFGFPWLNEFSAFITFNNAGDKVVNV<br>QEMVDTEFFQKFFPAYQSFLSQNK
**>gi|514825994|gb|AGO59036.1| PtaH (*Pestalotiopsis fici*)**
MSTNDEVFAKDNEFWKTYLRGRAQPPESFFERIFRYHEDHGGHFGTVHDCGAGNGPYSQK<br>LRSRFKHVIVSDVAPGNVELAKERLGNDGFSFRVARVEDFDDIPTGSVDLVFATNVMHWVE<br>PSRGAKAIVSQLKSGGTFIAAGFGPARFEDQKVQDIWTRISQSGGRRLIMKADDPTKILKVA<br>VRSSRYYDVAPTDTSLFVPGTQRIHLNMNNGGLTDIVYPEDYVAAAEPSYTGPQDDEIFESE<br>DGWSFETDLEGVKDHFATFPFSKEDPEVFAELWAELEKYVADGRPIRGCWPAKIILATRV
**>gi|514826008|gb|AGO59050.1| PtaR1 (*Pestalotiopsis fici*)**
MSSVPIDSVQAAQQVVSTKIKDKSWIMPTDPAEFLQQIAYQSQLLACLHWLGEFQILACVPL<br>SGSVPIKDVADLAGVPVSQLAHVIRFMATAGFMKEPRRGEVAHTPQSAAFVTDPSFLDAGIF<br>LAQVSARSARKMAQNSAISTLMGGDGANNGSDFDNGELLKSTSESPRVQREVTAYLHYVV<br>NEVSDTANLLAQLDWRKLGSSSVVEIRADRIYPASLVLTELHSTPRFTVQTFQEDSVEQGTA<br>VTTTATTSSSFISKSSEEPSPKRIKPGITYQKRSLGSPQVVTDATMYVMRLERPHSTSVQRSIL<br>DEGQIVSELRAHLGVLKHNSNATLILIGPLLPEAGAIDAKAEMVVRFRDLSLNQLTSEREME<br>VGELVDIIGDVQDESGCLVVVNKLYSRTSSTVALEVRYELYNYRKG
**>gi|514825995|gb|AGO59037.1| PtaR2 (*Pestalotiopsis fici*)**
MEAADPNNNLTITSPSTLLSNPTQPPAQPLKLRDSCHACASSKVKCHKEKPTCSRCRKRGIT<br>CEYFAHRRPGRKQENRAKDTTNHVERQENTTAVEMLDLNWPAPDFSTQTSIANDNLDVFH<br>DIFVPPDQLNNGLTDFTIDFDDFDIQSDPAEIASLPDTSSLESMFVTSPTAPTDTITPNVITPNV<br>GLSVLEGLPDTTHHTQAINLASYIQTPPTEKTPDSRIKHLEENKDPCMTRALSFLTQLSESTSR<br>ICKTSETGCSGTNKKSLPESLDGIIAENRRLLEAMSNILQCRCSEDDDLLCIQAIVASKILNLY<br>ASAIEIKPSPARVGSGVSTHTTAGQYEPQVEQQLSTRTHPQLASGRDPIRMAAQSVLGELHR<br>VQRLLSQMLQKSKDNETMRRKGSENGLRAVADKVPLTSGVSFGSIEADLRYKLGKLSIEIIT<br>LLRGA

Table S1. *Cont.*

<table>
<tr><th>>gi|514825996|gb|AGO59038.1| PtaI (Pestalotiopsis fici)</th></tr>
<tr><td>MPSPTNPAKVETPFTTAALGNGVDFWKAYVENRPHPSDSFFELISEYHHSHGDSAAQSAIAH
DVGTGPGNIAEKLLRHFDHVVGSDVNEQALAAAPALLPADSIKRMTFVKSSAEDLASANIP
ESVGKGQTDLILVSECIPLLDISKAFAAFRALLRPGGTLAIYFYSRPIFTGDNEAELNQLYDRI
ATRVCQFLLPFKGTPGFPIHYRAAEAMSSGLDSIPFDPEAWQDVVRYKWNADVPLTFNSKE
GYDFEVEPVDRRDHSTEITKEITDRDFWAEEWDIGRVASFLDSVFPNYRNKAGDKFEEVQS
LFTELETALGGPKATRKVSFPVVLLLATRK</td></tr>
<tr><th>>gi|514825997|gb|AGO59039.1| PtaJ (Pestalotiopsis fici)</th></tr>
<tr><td>MAASTAAQVQLSEEALGLARIFENPKGSLEAASKLLQKNHDEFHVFWRDVGGHNHIPHSVL
SILALGGGPAELQRAWDDGVAIQRPTPPLDEDVVKKLENPAEFRARIGSIPNYTNFLHFFRN
QMDKKGWQAVVSEYAFSRTPLAETIFAQLFEGAYHPFIHIGFGIEFNLPSIIAEGLAQAATHD
SAGIEGFFLEAERQAAQSKGPGKSLVQLLDEVRTTEKIKTAARLPDGPVRVRDGVIGRAGAE
IAALASQFRVPADQLSRGAAESINISAYTAGAAQRAGKARKIDFFHMHNTTSSLFLTVFLNQ
PWISTEDKVRIVEWKGRLDLVWYAACSAPDLNVDHVIGYKPAQSAGWGWKELYEAINVA
HDDGHLAKIVRALKNGEEVSRPFESGEGAEAFPIKGDSWLKLAQMSYDTTLDLPDDDKWI
WGAGFLPLWNKVPSL</td></tr>
<tr><th>>gi|514826010|gb|AGO59052.1| PtaK (Pestalotiopsis fici)</th></tr>
<tr><td>MKSLFLTGLLSALTWASEFDYLYTTEPVTPLPQGYPWGSKTANDSHPEGKLVTVHNDLEEG
TALHWHAFLQKETPWQDGVPGITQCPIAPGACFTYTFVADSYGTSWYHSHYSAQYADGIL
GPIIVHGHPTVPYDIVLEHLFDEDFAVVVSKSFADSRNATNIRQTKPANNNLINGRNSWNCT
LKDLGDDTPCQSNAPLSEFRLTPGKKHRLRILNVGGSAIQKFSLDGHKLQVIAHDFVPVLPY
EVEFLTLGVGQRADVIVEALANGTGTYTMRATIPPAPCANSVDHDATALVHYGNTTSTFSN
SSSEAWPSFIEALGVCDGLPTEEITPWYAIPAPEAPATTQIINVTLAQNETGQYLFYMDNSSF
RVNYNHPVLLLSNLGNNSYPDDPEWNVYNFGSNNSIRIVMYNNAIRTHPIHLHGHNFFVEA
VGLGEWDGHVDHPENPVRRDTAMLPQGGYMVISFNADNPGAWPLHCHVAWHVSSGFYV
TVLERPDEIAEYKIPSVVGQTCRDWWGYTNHTIVNQIDSGL</td></tr>
<tr><th>>gi|514826005|gb|AGO59047.1| PtaL (Pestalotiopsis fici)</th></tr>
<tr><td>MKHIVIIGGGFAGVSTAHRFLKNVGKSTTAPYKVTLVSRDSHFFWNIAAPRGIIPGQIPEEKL
FQPIAEGFSQYGPDKFEFVLGTATDLDVGGKTLVVDVDGKATRISYDYLIIGSGSRTKIPGPF
KSDGSTDGVKQTIHDFQERVKAAKTIVVVGAGPTGVETAGELAFEYGTSKKIILISGGPTVL
ENRPASVTKTALKQLETLNVDVRVNTKAKDPVTLPDGKKELTLSGGEKLVVDLYIPTFGVL
PNSSFVPSQYLDSNGFVQVDQYFQVKGAEGVFAIGDVSDSEAPQFWFVEKQSVHIAKNLILS
LSGKAPTPYKASATGMMGLQIGKNSGTGHFGNFKLPGFLVKTIRKTLFVENLPKTVDGSML</td></tr>
<tr><th>>gi|514826006|gb|AGO59048.1| PtaR3 (Pestalotiopsis fici)</th></tr>
<tr><td>MATPKRMWPTDLKHIVRYTSRRTSSRYRVPPGQKANPKLAPKVHAAASTPSTNTSHSTETT
PPSDNGFYDTAESPLSPNGAQHLTIATIPAEDSILLMHFLDKVFPLQYPMYRPDILEGGRGWL
LALLLQTKSLYHAALALSSYHRRMLVFERISEQCRATAAVQQEKHLETCLNEVRQAMVILD
QRTRQRKSYDGMGTVTSIVQLVFFELFAGQDHAWRTHLNAAIDVYDQNCRDKLEHLDLSE
ASKTILRNDQRLAVDGALVTQEVTTFRFMGGSIIWLDILSSLAAGSVPRLLSYHQGVLDAAS
QVKLENIMGCKNWLMCQIGRIAALQGHRRQDGWTSQRHGIKLHSIAADIKSEIESGMAREA
LESLNIQTSDSCGINNSSTNSVTLTTRMFAFMAIIHLHLVTHDFERLDNLRETIADAIRLLQSQ
VPCSMIPVIVAPLFIIGCVAAQGDEQSLFRASLASDTSQHRLYRHRKDVLSALEEIWSKRQTS
TDYTWNDVLDMGQHKYLLFL</td></tr>
</table>

Table S1. *Cont.*

**>gi|514826004|gb|AGO59046.1| PtaM (*Pestalotiopsis fici*)**
MSVPAQTSVLIVGGGPAGSYAATVLAREGVDVVLLEAEKFPRYHIGESMLASIRFFLRFVEL<br>EEEFDRHGFEKKYGATFKITEKNPAYTDFAASLGEGGYSWNVVRSESDEIIFRYAGKCGAK<br>TFDGTKVESLTFEPYPHEGFDESVHLANPGRPVSANWSRKDGSSGVIKFDYIIDGSGRNGLIS<br>TKYLKNRSFNQGLKNIANWTYWKGAKRFNVGEKNENSPLFEALKDGSGWVWAIPLHNDTI<br>SVGVVARQDAFFEKKKESGLSGEAFYKEYLKLAPQIKNELLRDATIVSDIKQATDWSYSAS<br>AYAGPNFRLIGDAGCFVDPYFSSGCHLAMTSALSASVSIQAVRRGQCDELTGAKWHTTKV<br>AEGYTRFLLLVMTVQRQLRMKDKNIISTDEEEGFDMAFKKIQPVIQGVADTRTEDEQTQRR<br>AAEAVDFSLESFEITPEKQAAVISKIERSQAEPELLEKLTPEEVHILGNIVNRTFEREKDELNL<br>THFTGDMIDGYSAKLEHGNIGLYKREKALLNGTASRAAAVLKSIHQVA
**>Afu4g14580 COORDS:Chr4_*A_fumigatus*_Af293:3847775-3849411W,<br>translated using codon table 1 (482 amino acids)**
MERQPKSLCDATQLLETANIISDTVQTIIAEWSAEAKAPQGSGKQNAPMLPSRELFDAQRTIL<br>AAVGKLTELVSDPSARILEVATQFQESRSLYIAAERRIPDLLAAGDEGGVHIDQISQKAKIEP<br>RKLARILRYLCSIGIFKQTGPDTFANNRISAALVSNEPLRAYVQLVNSEGFTASDRLPHTLLH<br>PDTGPSYDVAKTAWQNAVCTKKTRWEWLEERVAPEQLLESGGHYPGIPSLVMGLPPREDD<br>GLVARPELEIMGLSMVGGGRVFGTAHVYDFPWASLGDALVVDVGGGVGGFPLQLSKVYP<br>QLRFIVQDRGPVVKQGLEKVWPRENPEALHQGRVQFVEHSFFDTNPTEGADIYFLRYVLHD<br>WSDDYCVRILAAIRSSMAAHSRLLICDQVMNTTIGDPDLDSAPSPLPANYGYHTRFSHSRDI<br>TMMSCINGIERTPAEFKGLLQAAGLKLKKIWDCRSQVSLIEAVLPEMNGFR
**>Afu4g14530|gstC COORDS:Chr4_*A_fumigatus*_Af293:3835440-3836117W,<br>translated using codon table 1 (225 amino acids)**
MPDIQPITVYGKGGPNPPRVAIILAELDLPHKVIEVPLSKVKEPDYVAINPNGRIPAIYDPNTD<br>LTLWESGAIVEYLVSHYDPDHRISFPAGSNLAALATQWLFFQASGQGPYYGQASWFKKFHH<br>EKVPSAIERYVKEINRVTGVLEGHLSRQKVAADGDGPWLVGGKCSFADLAWIPWQVIVTAI<br>IQPEDGYTVEDYPHVKNWLDRMMARPGVQKGMADIFPST
**>Afu4g14510 COORDS:Chr4_*A_fumigatus*_Af293:3833644-3832621C,<br>translated using codon table 1 (282 amino acids)**
MLEKVFHEKSFADQYTYGAKISELYAETLITESGIAKSHQRPLIILDNACGTGSISSTLQRTLD<br>ERNKRSLKLTCGDLSEGMVDYTKQRMQAEGWNNAEAKIVNAQDTGLPSDHYTHVYTAFA<br>FNMFPDYKAALRECLRILQPGGTLATSTWKTANWCTIMKPVIATMPGQLSYPTMDEINTML<br>NKGWDRESDVRAEFEQAGFDHVNITTVEKQCLLPVQEFGEACKILLPYILSKFWTQEQRDQ<br>YEADVPSYLMRYLEREYGKDGLAPMKGVAIIASGRKP
**>ATEG_08460 (conserved hypothetical protein)**
MLASMRFFLRFIDLEEQFDAYGFQKKYGATFKINSKREAYTDFSASLGPGGYAWNVIRSEA<br>DDLIFRYAGEQGAHIFDGTKVDDIEFLSYDGADGANFTPAAFLVNPGRPVAATWSRKDGTR<br>GRIKFDYLIDASGRAGIISTKYLKNRTVNEGLRNIANWSYWKGAKVYGEGSDQQGSPFFEA<br>LTDGSGWCWAIPLHNGTLSVGVVMRQDLFFGKKKAAGSPGSLEMYKLCLQSVPGISGLLE<br>DAEIVSDVKMASDWSYSASAYAGPHFRVAGDAGCFIDPYFSSGVHLALVGGLSAATTIQAV<br>RRGETSEFSAAKWHSSKVTEGYTRFLLVVMAVLRQLRKQNAAVITDDKEEGFDTAFGLIQP<br>VIQGQADTGESEQQRMVAGVQFSLERFGQATPEAQRALLDKVQFAGQNAEELEKLTADEL<br>AVLHNIIGRQLKMTKVEKNLDNFTRDVIDGWAPRVERGKLGLQRADTSIMTAEMKDLFQL<br>NRSLDSTKAGIQLPA

Table S1. *Cont.*

**>AN10049|mdpB COORDS:ChrVIII_*A_nidulans*_FGSC_A4:4457666-4458357W, translated using codon table 1 (214 amino acids)**
MTLQPTFEGRTPEQCLNVHTDSHPDITGCQAALFEWAESYDSKDWDRLKQCIAPFLRIDYR AFLDKLWEKMPAEEFVAMVSHPHFLGNPLLKTQHFVGTMKWEKVDDSKIVGYHQMRVA HQKHLDSQMKEVVAKGHGHGSATVTYRKINGEWKFAGIEPNIRWTEFGGEGIFGPPEKEEN GVAADDQVMNSNGSSEVEERNGHVVNKAVEVRSV
**>AN0146|mdpC COORDS:ChrVIII_*A_nidulans*_FGSC_A4:4456446-4457370W, translated using codon table 1 (289 amino acids)**
MSPAIQRLSLVSSHLNSNVSALPKMTATTHAPYRLEGKVALVTGSGRGIGAAMALELGRLG AKVVVNYANSREPAEKLVQEIKELGTDAIALQANIRNVSEIVRVMDDAVAHFGGLDIVCSN AGVVSFGHLGEVTEEEFDRVFSLNTRAQFFVAREAYRHLNTHGRIILMSSNTAKEFSVPRHS VYSGSKGAIESFVRVMAKDCGDKQITVNAVAPGGTVTDMFYDVAQHYIPNGEKHSAEELQ KMAATVSPLKRNGFPVDIAKVVGFLASREAEWVNGKIITVDGGAA
**>AN0147|mdpD COORDS:ChrVIII_*A_nidulans*_FGSC_A4:4454244-4455887W, translated using codon table 1 (521 amino acids)**
MTHFPVNIASDKQEFDPERWAKTPTTESSVNGENGTAPTSGLPSRHPSTGISVLIVGAGMGG LMTALECWRKGHDVAGILERSEGPVYSGDIIVMQPSAVSIIRHWPDMLHDMKAEQVHAVV SYETHDGRHIYGPTVPSFNDPEHLETRKGPFVAPAQVRRKFYRMLLRQVARCGLRVEYGKT VKSYFEDEKDGKGGVIIATTGEAEVRVADIVVAADGLKSPSEILIAGQHVPPRSSGLSIYRTA FPKDLAMQNELVRKRWSDSPPIWEYWLGPGMYLGVFVGDDIISFGFTPRDDIVEGTATESW EPDTDPETVAQAMLSGAGDWDPAVLALIRSAPKGAIVHWPLLWRDLRREWTSPAGRVVQ VGDSAHSFIPTSGNGGSQALEDAITLATCLQLAGSSQRAYLGTKIYNLLRYERVSCAQKMSF VNSQLKTGTDWDAIWKDPAKIRTRFPKWIFQHDPEAYAYEKFGEAFAHLLDGREFVNTNY PPGHEFRAWTVEEVWRNIADGKRVEDLLDGDWS
**>AN10035|mdpI COORDS:ChrVIII_*A_nidulans*_FGSC_A4:4441618-4439762C, translated using codon table 1 (592 amino acids)**
MSVSRSCFRPFLPAEIDGGHLPVDPSVFTHIERGLHQNPQGFAIQSTHQQPCHFSALVQTGSG TENGGAPNYDAVEREPGTCLAWTYTQLHHAALRIAAGLLARNAQPSTRMLLLIPNGAEFCL LLWTAVVLRVTIVCLDEELLNVEQHDELRRMLKTINPRVIVVQDVKGADVIDVALRNLPLD PDILKITLSELAGSQPDSAWRSLLSLSLTPALSASETESLLSSARWDSSNAARTYSILYTSGTS GVPKGCPLHISGMSYVLQSQSWLVNAENCTRALQQAHPCRGIAIAQTLQTWREGGTVVMT GNGFNAGDLVHAVKRHAVSFVVLTPAMVHPVADELKGRNGAADSVRTVQIGGDAVTRGA LEICTRLFPKARVVVNHGMTEGGGAFVWPFNRPRDIPFYGEMSPVGSVARGAAVRIRGANA TVARGELGELHVSCPSIIPGYLGGVSAQSFHDEDGRRWFKTGDVGLMDKQGVVFILGRMK DMINGKVMPAPIESCLEKYTSVQTCVVNAGGPFAVLARYTGKKEAQIRRHVVRALGKSNA LNGVIYLHQLGLERFPVNGTHKIARGDVEGAMLAYLQTEPTSR

Table S2. The composite multifasta file used for the group V2 MultiGeneBLAST search. This list contains one member of each group of orthologous genes in all group V2 clusters.

**>gi|297242539|gb|ADI24939.1| VrtR2 (*Penicillium aethiopicum*)**
MPSLSSKTSTMQRSCRPQMSACPNQQQKDRPVPQLSCVLCRDRKLKCDKLDPCSNCTSSGV<br>ACTPIYRPRLPRGRHARTVQTKASTPPDTRRRGSSNESTTAPAPDDGGLGTHIDQLDNLVQD<br>REVSKLGLSGEGNGLQELISLVSDETMPATAWSTHCFGTISRILSSRIRRLESLVQETARIQTP<br>KRARKPMTPVVVQWYSAPLAGCNWNRMVVQTPQGLEVQQFPAPPTSYSARRSPELSGNDI<br>WADLMDHDMHDPPQYNALELPPDLTNEGGVDNMGSSGRDDPINNGFNALRLLGINNSLSP<br>SFISLPRDRLSASKLCQVYLQNVDPIIKILHRPSLSRWMVDGAPTYLGSSEDDYAVKALESA<br>VCYTAANTMTEHQCQAAFQKTKSSIMAVRRKMCEDALENAGLLTTRDMTVLQAFILYLVT<br>PTDLSKIGRRSEDKDTAVWALVALAIRLIKAMGLNQEPSEGARKGESFFQQQLRLRLWLTA<br>CLIDLQASFAQATDPLITHRDAACAVPYVANINDSDFDVDTAHPVASHEELTDTTFALVTYR<br>VQVAGRLFNFGPGCSTAAERHKLAQEVQQQVFTLLHYCDPESSSYAWFTWHSTQSIIFAVR<br>LSELLPFRCGQPGGHVPPPSPRAEGDTTLLWRALQNLEKAQLIRADPRGDGFRWYITTPWL<br>ALSTAISECNSCTDVALVCRAWPVIEISYRQHEELQISDECQLPQGPLVHLMNKTREKLAPLL<br>QEGGARLSDSQTVDRASADSLQPPVPVGSIPIDPLLTNGSLGADTAMSEASSIGSLPPFEQQC<br>WKQMTMPTDGAPVRDGVVFTSELYNPLQSDFLNSHG
**>gi|297242538|gb|ADI24938.1| VrtL (*Penicillium aethiopicum*)**
MSKLSDNHSSASEGEKEAGDLESGPTAISSEPSFDDADRDPNLITWDGPKDPENPKNWPKGL<br>RWKNTWTVSLFVFISPVSSSMIAPAMSDLAKSLGMHAEIEIYLSLSIFILAYSIGPIFFGPASEL<br>YGRVRLLQISNVWYLAWNLGCGFATTKGQLFAFRFLAGIGGSAPLAIGGGAISDMWTAEER<br>GKAMGVYTLGPLLGPVVGPIAGGFIAEYSTWRWVFWSTSAAALAVQVVGFFWLQECHPGT<br>LLRKRRDRLAKETGNENLHTAEKVETLGYKLLHAFERPVKMFTTQPIVFCMAIYMAYLFGI<br>SYLMFATFPEIWTVVYHESPGIGGLNYLSIAIGSFIGLFFNLKLVDRIYRSLKARNNGVGKPE<br>YRMPSLAVGSVISTIGLFWYGWSIGNTHWIMPNIGALIFAMGTISCLQGMQTYIVDSYQTYA<br>ASAMAACAVLRSLCGFGFPLFAPYMYNSLGYGWGTSLLAFITMVVGWGAPFAFWHFGPRL<br>RAMSKYASG
**>gi|297242537|gb|ADI24937.1| VrtK (*Penicillium aethiopicum*)**
MAFSTYLGSLESSLVLKGLAGVWLVWYIGRVFYNIFLHPLANVPGPLLCKFSKIPWDYWQ<br>WTGRLPQNTAKVHAKYGEIVRIGPNELSFTNNAAWNDIFAKVPGRAQWPRHPKRVPQGKN<br>GPQSIMNTAGTYHARFRRLLNHAFSEKGLQEQQDLITKYIDIFVSKVDGFARTGQSLDVTK<br>WFVMVGFDVISDLGWSEPFNCVENGEVHEWMKTFAETAFDTQLKFLFRERGLMFLAPYLV<br>PMKLQLARLNNFKYARARVEERIKTGGTRGDFWDKISVKSAGDNASGEGLTKEEMVVAA<br>VTLVGTGSHTISTLLTGLAYFLGTNPHTMKKLVDEIRTSFNSPEEIDLVSVHKLKYLTACLNE<br>TMRLYPPVINMLWRTPPQGGGHASGIFIPEGTGCNMSFFGIAQNPDYFTRPLDFCPERFLPDP<br>PAEFRDDNHEAYHPFSLGAYNCLGQNLANAESRLIMTKLLWYFDFELDGTVDKDWLDQKS<br>YGVFIKKELPVKFHPGPNAVRHVANGNGVATNGHANGHANGHARINTK
**>gi|297242536|gb|ADI24936.1| VrtJ (*Penicillium aethiopicum*)**
MTNSPIADLVHHPERVQSPSLVNSKMNGDAKAVTEWTEPGPAAFDFRSDTVTRPTEQMLA<br>AIAATTLQDDDFRQDPTTLGLEAWMAELTGKAAGLFVVSGTMGNQLGVRAHLQSPPHSVL<br>CDARSHLVTHEAGGVASLSGAMVSCVTPVNGRYMTQADLEAHVNRGTLITDCPTRLVVLE<br>IPLGGVILPLDKCRRISEWARAQGIALHLDGARLWEAVAAGAGSLRDYCACFDSVSLCFSK<br>GLGAPIGSVLVGSETLRERARWIRKSIGGGMRQAGVVCAAARVAVEATFLGGLLKRSHAR<br>ARDIATFWEIHGGRLTYPTETNMVWLDLEAVGWTPERLIRRGAELGLRFMGARLVVHYQI<br>GDEAIGRLQDLMLEILVSGLVDHPRDS

Table S2. *Cont.*

**>gi|297242535|gb|ADI24935.1| VrtI (*Penicillium aethiopicum*)**
MGARSKEARHRWFSRLPPDASSSFLPCKRPKSVAFVLWIRPSCFLRESLPAELSGLPGLPLPP<br>QAPTNTMNCGLPEAALSVIDWGRLKSGDVNEGARLLSACDDQGFFYLDLSSEPSFLHDHKS<br>VLHFMDQYFHQGLADKMKDDRQSDTHGYEPVATSTGALNTLPDYYESLKASRDELHGDG<br>RNLAPAVCDRQDLFLRFSDMMHQMVIAILQELDCQLGFGGDRASFQDFHRKDAESLTTLS<br>MFRYPKQETLDLGVGHNKHTDIGTLTFLLCDQWGLQVLSKDPAGWRFVAPREGHAVINVG<br>DTLRFLSGNRFRSAVHRVIPTQRLQHEDRYSIAYFLRAANDTQFTDSAGRQVSAKQWHDEK<br>FDVFRETHEEQEKMPILTGGMERLENLPGIWCGVRLTDGICHRLGAEDC
**>gi|297242526|gb|ADI24926.1| VrtA (*Penicillium aethiopicum*)**
MLHPTEVEKFPPTLLYFGNEFPNDDVNELFRRLQQHSKDRRFRLLNAYLEESILVLQDEVAK<br>LPHHIKSRVPYFDNIVTLSEHGYLRDLGLGAAMESAFLLILQLGLFIGNHEAVDRELNLPKN<br>VTTVAGLSVGLFSAAAIALSASLAEVVRNGAECLRVSFRLGVYAGDFSSSLEAPQPEGMLAS<br>WAHVVTGMTEESVQSELTRVNEDLGNPETSKVFISAADKSSVSVSGPPSRIKAAFLQSSDLR<br>YSKSLPLPVYDGLCHATHIYSQDDVNTVLEISESLIPATRPFQLSVISSRTGVPFTATTASDLL<br>SEIATELVMGTIYLDNIIEGIVRHIGAFPGAQSCRIDSFRTSIIFKGILEAIATDHPDLTIEKNDL<br>VDWVHQDFGTRRRNDPANSKLAIVGMACRMPGGANNVEEFWQLLEQGRDACTTVPPDRF<br>DLETHYDPTGKTENAAQTPYGNFIDRPGYFDAAFFAMSPKEAEQTDPMQRLAIVTAYEAME<br>MAGLVIGRTQSTRRDRIGSYYGQASDDWRELNASQNIGTYAVPGGVRGFTVGRINYFFKLS<br>GPCLCIDTACSSSMAAVHAACTALWAGDVDVALAGGVNIITDPDNYAGLGNAHFLSPTGQ<br>CKVWDKGADGYCRAEGIGSVVIKRLEDAEADNDNILAVVLSAATNHCADAISITHPHAGH<br>QKDNCRRVLRKAGVSPMQVSYVEMHGTGTQAGDAIESESVLDVFAPLKPLRRPDQRLHLG<br>AVKSNIGHGEAAAGISSLIKMLLMFQKNAIPPHIGIRTEMNPQLPKDLGRRNAGLVFETTPW<br>LRPEGKKRISVVNSFGAHGGNTTLLLEDAPERHRQRISPESTDGRSVYAISVSAKSKKSLQG<br>NLSSLLGYLEQHPDTDLADLSYTTCARRTHHNLRVATVVSSVSALQKFLRSAIDSNIATTVQ<br>SVPSNIPSVVFTFTGQGASDRGVRQELFDDFPAFRTQVLQLDQLVQRLGFPSVVPALRGSTD<br>EEVLSPVVSQLSIVVLEIALSRFWRLLGIRPSAVIGHSLGEYAALEVAGVLSAADVLYLVGRR<br>AQITEQRCTPYGHSMLSVLATPDEIDRVVRRVPETSNVEYEVSCQNTHEDTVLGGAKADIE<br>AIRKVLETTSYKCVPLAIPFAYHTSQMDVVVDELEEIAKNIPFKAPSIPVLSTMLGTVVFDGK<br>TINPTYLRRQTRGTVKFVAAVETARDLGLIDEKTVWVDLGPHPVCVGFIRKLSPESRIAASC<br>RRNEENLSTITKSLVTLHLAGATPLWNEFFRPNEQVYRLLNLPKYSWNETNYWIPYLGTWA<br>LDKALLKYGITPVGAKAPATLPAAGLRTSTIHQTTLETIDSMTATLHVLSDMQAPEFRAAVY<br>GHTMNNCGVATSSIWTDMALAVGEYLYRKLVPQAKEVHMNVCDLEVLHAQVISKVKGCS<br>QPLALEAHLDLDMQYMSLKWYNTNAATGERAPEWFASAAVRFENPDAWTAEWNRTGHL<br>VLGRIETLRRLAADGVANRISKRLAYTLFKNVVDYSDWYRGIDDVIMNDYEAVANVTLIPD<br>RHGTWHTPPHWIDSVCHLAGLIMNGSDASNTQDFFYVTPGSDSFRLLKPLAPGAKYISYVR<br>MFPLSAEAGNMYAGDVYILKDDVIVGVLCQIRFRRVPRLLMDRFFSPPTADNAGVHGTPGS<br>QRSQAPPAATHAATKSPQKTLQVPSGHVPNKASIVDTNHVQASHPSAVPVRHDQSNGVSG<br>VSDSDSSTSITSSNSTADTSTTPTESEDADSGLVGQCIKIISRETNLDMSELTPDATFAQLGVD<br>SLMSLVLSEKFRNELGIDVKSSLFLECPTIGEVKEWIDQNC

Table S2. *Cont.*

**>gi|297242527|gb|ADI24927.1| VrtB (*Penicillium aethiopicum*)**
MAPYLQHFIAWFGKDSILSKLIQIFNPPSHSDSAKIIISDDDGRSPEPPGGEEILWRHSAPKSTQ<br>MWAFLQKVNKKYGYSLRTYQDLHHWSITHRGQFWGEAWDYCGIRHSRQYDEVVDEAAA<br>MWPRPAWFRGARLNFAENLLFPKVPQAVSDEAIAVISVDEGGKRDFITWQDLRERVRRCQS<br>GMRALKIQPSDRVAGYVANHSNALVAMLATASLGAIWTAVSPDTGVIGAVDRLVQIEPRLL<br>FTDNAVIYNGRIHPVLTKTQEIITALPSLEAVVVMRTVAGVQEDLPSSPRSPAKRLTYNSFLA<br>QGPETQKLEFVHMPAEHPLYILYSSGTTGPPKCIVHGAIGTLMQHKKEHMLQSDIQPGDRLC<br>FVTTCMWMMWHWLVSGLASGATVVLYNGSPFYYAPSSHGTSAKDDLAMPKLVDELGITQ<br>FGASATYFSMLERRKHLPRSLTQGRLSLETLKAVYSTGSPLAPSTFRYIYQAFGSQVNLGSIS<br>GGTDIIADFGVPSPLQAVVAGEIQVIALGMAVQAWGPTGMDLSVTGEPGELVQFWGPSGAS<br>KYESSYFAKYPGVWAHGDHIQINPRTGGLIMLGRSDGTLNPKGVRFGSAEIYHVLQYHFAA<br>QVEDALCVGRRRRQDTDEMVVLFVKMRQDQLWSLALAEAIRRTVREELSPRHVPELIIECP<br>EIPVTANGKKVEVLVKRIVSGVEVPAAGGSGTVNADCLQWFQEWATQN
**>gi|297242528|gb|ADI24928.1| VrtC (*Penicillium aethiopicum*)**
MGDATATEVFPVMDSIAVAMGDVESQLRVFHNVSRFLPTDDANQVFWWRTTGRHFAIMM<br>HEARYSEARQVELLLFYRFVIAPRLGPRPTSATPWFHSRVAPGIGDGSPIGYSWRWGTGPDT<br>KPLIRHYIEAIGPLTGTTADPLNEFAAKEMLYQLGQLVPGVELPLAWKFAAHIRPSLTDEPTR<br>AVAGSSILIGLQCAPESAGIEVMAGLMTRSPAQVPELLHSIFPRAMRDAYGPDASLDGLNMV<br>RDFVCHDPQGQYLTILGTTAIDCCAAASSRFKVYVTTTNTSFAHLAAVMTLGGRKPEAPES<br>LTQLQELWYALKGLDPEFPVTAEPLSSVCGAANGTASGNPNANVSGVTFYFDIHPKYPFPHI<br>KLQVDISKHTISDLGAINAVTEFLARRGQAADAQAYLNVVRAMVPDEELRTRRGLQAFFAF<br>AFKNGAVDITSYFLPQIYRRYAEVQAELEPRKDCQGRSELSSKLQRRSRFDSY
**>gi|297242529|gb|ADI24929.1| VrtD (Penicillium aethiopicum)**
MATSTTTSLKEFLSVFPQLVADLRALCLEEYQLPACVWDRFESTLNHNTLGGKCNRGLSVI<br>DSVRLLRDGLELSPAEYFDAAVLGWLVELLQATMLVLDDIMDGSPTRRGKPSWYRVPGVG<br>MAAVNDATMLESAIYMLLKKYFAGRAIYLPVVDLFHETALQIELGQAFDMLIANEGTPDLT<br>TFVPATYSQIVTYKTAFYSFYLPVALALHAVDAATPTNLAAARAILVPMGEYFQVQDDYLD<br>CFADPTVLGKVGTDIIEGKCSWLVVQALQRASTDQAQLLAENYGSASGESSVKALYSELDL<br>ESVYRTFEEQRVAELRTLITGLDESQGLRKSVFEELLGKIYQRRK
**>gi|297242530|gb|ADI24930.1| VrtE (*Penicillium aethiopicum*)**
MFIAAVTHNWMERLAALSLLHYVLGAIFLLLLFHMLSNFFHPGLVDVPGPFAAKFTDLWRL<br>FKVWQRRFKEDLPGLHASHRSTLIRIGPRMVSCSDPRAVELIYGFHTEFSKSDMVKAMAPIY<br>KGKKQPTMFAAADNKTHARIRKPVAGAYAMTSIMQRMDELFIRPKRACDIHNWVQYFAFD<br>MVLEMTMSRNLGFMKAGGDVDGVLKQLQKDLDYRGIALAMPIIDRIWRLNPVSKFFKPKQ<br>SGHFAMRCKRILEDRMAYEKSLDSRTQQQQDQKPHDFAHRFLEAQRKDPSISDGQLIGYMQ<br>ANLIAGSDTTAVVMRTAIYYTLKQPWILQRLVTELDQYHGPLPVPFRIARFEMPFCGAIVRE<br>ALRRHFAFIGMMERQTPPCGVVMPDGRRLPGGVVIGMHGDLIGRDRAIFGEDADEFNPLR<br>WLARPGEPEAKYQERLRAMNAHDLAFGHGPRGCIGKHVAEMEIYKFIPTFFALIQPRFMRP<br>EQSWTVRQLFVFKQSGMDMMLDWRQGKGLQSMA
**>gi|297242531|gb|ADI24931.1| VrtF (*Penicillium aethiopicum*)**
MTTTTTTDDTQKLDPSASDEVIYKSWDLLIYEIWVLGIVSTWAWGCSTTEYLLPQFRANVG<br>TNHLDVGSGTGYYLRKGGIPASTRLTLLDLERPALDLGLQRCGRSDARGLQADILQPLPVID<br>KFDSVSMYYLLHCIPASVEDKCAIFKHIKHNMTPDGVIHGANVLGKGVRNDGHFAAYVRR<br>GVLKAGIFHNLDDNAYDFEHALRMNFEEVETRVVGSVFIFRASRPKLDEGDLLET

Table S2. *Cont.*

**>gi|297242532|gb|ADI24932.1| VrtG (*Penicillium aethiopicum*)**
MATRIPFDESYWEEYLSGQEASLPALPAVTQLSPRVTRLLAGNPGIMQLQGTNTYLVGTGP ARILIDTGEGRPVWHATLAEHLRTHHLTLEYILLTHWHGDHTGGIPDLIAHDPTLQSRIYKH HPDRGQRPIRDGQRFTVTGATVRAVFTPGHAIDHMCFLIEEEKALLTGDNVLGHGFAIVQDL AEYMASLARMAALGCERGYPAHGAVIENLPAKMQLYIHHNEVRVQQVITALASVVKLPGK RVGMTVPEIGRAIYGEVPREIVENAIVPFLSQVLWKLAEDRKVGFEPGEANKRRWFGLVTQQ
**>gi|297242534|gb|ADI24934.1| VrtH (*Penicillium aethiopicum*)**
MQRANHTRPVLIIGAGLSGLAIGRLLTNNGIANIVFEASPPERSQGFAISLHDWGYSLLLEAL GGLSLRAMTKAVAPDRFIGGTGWVDLIMRDNTTGKVLVEPDVDARPAVIRANRNSLRAW MADCGDDELDVRYGHRLKSISGSVGNVQAVFENGAEYRGSIVIAADGVHSAVRSQVLPHIV PEVLPVVVYHGEFQVSHDEYDRCVRPVIGTANILAGVGDGFNTPITVCNITKTQVHLDWSY SRPARGENDPLFSTKTPEDQTRDLPQALLEELASRQLAEPWAKYINPETIQQHSVFRWISRCV YMPTADALHAAQAGVVFIGDAWHAMPIFGGEGGNHALVDSVELAAAMVKEANVERAVA VYYEGAARRCQEAVRRSRSRFYVLHRPMAEWRDIAEKRRAKAALEQKH
**>gi|297242533|gb|ADI24933.1| VrtR1 (*Penicillium aethiopicum*)**
MEDTTETTETTDTTAVSRLVPLAPAPARAPSMDAVNGSFDSELTTARRFNCQSCVRKKIKC NRAVPTCASCSKAKLHCVYQSRPPRKRKRSRGEEDVYERLAQYERILHDHNLLQAAAASTP SGRDTETSAISTRAPTPVLPDAQTHTKAGKVLLSTDGRSRYIDNVLLLDAGEGDLCELPESE QEDYNHDETSPDESTPTGLLGALAAHTISGAIIGNTQSLTNLHPTYEQAAKLWQAYVKNVE PLCKILHVPTVAKMFDTVSKQPAAVSKNDECLMFVIYYFAVFSMSDDECLHEFNYPRAQLL SRYQTTVIQALVNASWLKTTAMPVLQAYTLFLIALRTQIDSHTFWILTGIAVRLAQRMGLHR DGESLGLPPFEVQMRRRLFWQLLPLDSYAGQTSGTGISISPSSWDTKQPLNINDDQIFPGMTQ PPCEQRGASEMIFCLSRMELSNFYIRTGVKLKEHGDTIQFRDAEDIERLIDEVEDLIETKFLRY CDILNPLHFLTTGIVRSAIDAVRLRARMPLLKQQTITDAQRRRLCALAEKVLDTNSTIFSNPS TQNFRWQMQAFFLWDALLCILRNIAEVGFYSPSELAAAWSKVANVYANHDELVKARRTLY VTIAKVTLKAWLANPPRDSSPQPAFITALLTQHEPKGINQQQNSVLSDDKAADGASLFDEFF DNMNGTDLDINNAFNLDSSSDWLFWDQICRGTSLS
**>Afu7g00180|nscE COORDS:Chr7_*A_fumigatus*_Af293:44976-44029C, translated using codon table 1 (315 amino acids)**
MHIFITGATGFIGRVVSELAIQQGHTVHGLSRSPQGDEILTSIGAIPIRGDLATHNILREQSAKA DAVFHLAFDHDFGKSYDQIIKLDTEAVDALAAPLVGTSKPLIAASGILTVRPDQGDCVVNES APYTKNTRVRRHVCEENALSWAERGVRVNVVRLPPYVYGRANETGFAARMVRMAVDNG VSGYIASVKDRCVTSVYVDDAAALFLLLASDKTVKAGEIFHGTADWDTTYGMLAKAIGRA VGVPVRQFEREEAEERWGAFLSSFFGLIIRASNGKAVEKLGWKPSGPSLVEELETGSYRQVA ERFKQMKN

Table S3. The composite multifasta file used for the group V3 MultiGeneBLAST search. This list contains one member of each group of orthologous genes in all group V3 clusters.

>gi|297242553|gb|ADI24952.1| GsfR2 (*Penicillium aethiopicum*)

MPPLYRRSCITCVQSKRKCDQGLPKCQRCLAKNIHCEYNPRYPNRRRQTTERNVDENVSLVEPIAEE
PSRGCQLQRSPARPTSPTHSPHANDIFFNFANDPFNLESIPQDNFLNSTIFEDVVTQQAPNDTERITSDT
TAQARVEFAAKKLSVIPKIFSQQGQTMFIHRQLFQDRAPPALQDALSACALYCLKSTENQTLVFRNLE
HKRKQLISSIDPLLASKLDLLEALQALVLYQIISLFDGDIRLRAQAEADEPVLLMWAAQLTLRTPQFQ
PPLGLSNPQSLAGSASMDWGRWLIEESSRRTLITASMLKGVYSFVKLGYDTVPDMRMSFTAQAVLW
NSQSEISWRRAYKEKERLEIQVTHWDETIAKAKANDLEELGVLIMVMLKGTGATGEWLGHSQNIRY
GLEEAYYGSV

>gi|297242552|gb|ADI24951.1| GsfK (*Penicillium aethiopicum*)

MPATVLITGGNRGLGKGLVATYLSTPDTTVIATVRDPSKCESLSALPKALGSNLLLVKLEVTSKDSIT
TAIGTLDTHNIGSIDVVIANAGISGPTSSLAEAPVSELQRYIDVNAYGPFELFKAVLPLLRSSNSGNKA
KFVCISSAGGSLAAMYNFMPISAYGASKALANFLVKWLALDNKDIIIWAQNPGSVDTDMARDGLDL
AKSLGFDLSSLSFTSPEESACAIKKLIDGATTEMSGKFLDHDGSELAW

>gi|297242551|gb|ADI24950.1| GsfR1 (*Penicillium aethiopicum*)

MSDGPETAEGDTDDAVQDAAVNSRVASESSARSQPRATVVGCLPPTPFAIHTTPAPSEHSKEKNVSR
RLPTEKTPSRLATPQFPPTPVSSRGSIAEPSAYPSITQALRSCLPPQKDIEILLSNLSSMSIFCYKSSFKLC
SSWPSEMTEEQIPIANLLYSETHPVLLARHMLLFAVGLQHLSPTKAIPGLTRHHRAIMEQLADSAIKL
VNTDDVLLGTLEGLENLILESFYHIDGGNIRRAWITMRRAVMTAQLLGLHRPGHYRFKTVNKQNDL
DPAVMWACIVSTEQFLCLLLGLPTSTSGASFTIPRATSACVESGNLPVLIPDVVRKIIERNQTHVPQEA
LDMTQKIDHELLGVVKQWPPAFWRPLQLSGLEVDSADAFWETRRAWDHIFYYSLVNQLHLPYMLN
PSHVSQKVYSRIACASASREILIRQIAIRTFNPVTAGCRMGDFVALIAGMTLMLAHILSHCSKGTENLL
VHQRVGDRATVERALECMESMSEQHEDILTAKCAALLKNLLDIEAGPAEARSDDGQKDDQNVLVV
KVPHVGAIKIARDGISITPFDTEQEQVSHDGVTIGGFGSIHVSTPHDSDRDGDHQADVVTPNDTASQA
TTQVVRPASARKQWRPVSQRSSGDVFTLPEETFPDASAGMEEWLFQGLDTAFFDVLISGAGEQPLNS
TDTEGWNFVMSP

>gi|297242550|gb|ADI24949.1| GsfJ (*Penicillium aethiopicum*)

MDKQNTDSEKRATAEAPPQSLCTLVSSSEQGGMDITNHNAKAGAADEYPHGVRLAAVVFSLMLGM
FLVALDNTILGTAIPKITDEFHDLNKVSWYGSAYLMTFGCGFQSTWGKFYKYFPIKVWFLVAVFIFE
VGSLICAVAQNPTTLIVGRAIAGFGGSGVGVGIFTIIGFAAPPENRPQLLGFTGATYGIAAVLGPLIGGA
FTDKCFYINLPIGGVAAGTIFLLFKPPTSASPAKATPKEKFLQMDLVGATLMMGLIVSYILALQYGGQ
THSWKSSEVIGLLVGFFLFVLAFVTWEIYQKERAMIVPRLFMRRYISVGSIYMFFFSGAYFIILYYLPIY
FQSVYNSSPIGSGVKMLALIIPLTLAAIVQGWALSKIRIVPLFWIIGGALGTVGCGLFYTFDTETSVGK
WVGYQIIVGFSTGWTFQIAMSNAQVHAPPEDMSQATAIVNFFMTVGGAFFISAAQCAFSNQLIKTITK
NLPELDPTVAISTGATQIREAFTASQVPIVVDAYMVGLKAVFAITIAAFGVATVIGFFGSWKKLLADE
LEKATGGVA

Table S3. *Cont.*

**>gi|297242549|gb|ADI24948.1| GsfI (*Penicillium aethiopicum*)**
MAIPQSCTVLVAGGGPGGSYTAAALAREGVDVVLLEADCHPRYHIGESLLPSMRYLLRFID<br>LEDTFEQHGFQKKLGAFFKLNAKSAGYTDFIRANGPNGYSWNVVRSESDEILFRHATKSGA<br>KTFENVSLKSVNFEPYENDKFTSQDKLTNPGRPVSAEWKTKDGCSGTISFDYLVDATGRVGI<br>LSTKYLKNRKFNESFRNIAMWGYFKGNIPPSPGTDRENQPISEGMRDGSGWVWMLPLHNG<br>TVSIGAVVRKDIFQAKKKALPEGTTEAQTLASLVALCPTISSYLEPAELASGIRQAADYSYSA<br>NAYAGPNFRIVGDAGCFIDPFFSSGHHLALSSALAAATSINACIRGDCNEFDASRWFAKKVD<br>EGYTLFLVVVMAALKQIRMQEQPILSDLDEEGFDRAFTILRPVIQGAADKETAPKAKGESIT<br>ETIDLCLTALNDLHDTELQRKLTSIVEAKGTPEQEQLLGKLSPDETAALHRMRAMHSILPMG<br>ELEDFENSNIDGFKARLEKGSLGLRRERALCRDHAGDLQM
**>gi|297242554|gb|ADI24953.1| GsfA (*Penicillium aethiopicum*)**
MTSAKVLYFSGEIPQGDPEGDQRTLFRKLHLLSKERDHVVLASLLECVTLTLKDECSKLSPQ<br>YRDLLPPFESVLDLTDHVVQLRKTPLGGAIERVLVLVFQLGSLVAYHEAHPLEYNFTPASTV<br>IIGRGSGLLSAAAIGLSPSIVMVPSIAKEIARISFRFGLVVDKVCRSLEVSSDEINSDGAWVYC<br>VHGIGEKEARDAVNQFNEIKAYPSTNGASVFNVDDAGNSVSIGGPPKTLEALFSESNIFKKT<br>KNVAMRKIQGMWHTDRVYGPEHVEQIVPKIESARELHVPLISPVSGDPFRETEAGPLLEQIM<br>EEILMERVRWDMIIETVSKQLKQLMPKSVQLVSIQPSHYNQNMLERWKSELPDAAVSGLTM<br>MPAILELALEQSPPKDTRSSKIAVVGMSCRFPGSDTTEEFWERLMLGEDMHRHIPPDRFDVE<br>THVDPTGKRHNTSKTSYGCFVDNPGLFDAMFFGMSPREAEQTDPMQRLALVTAYEALEKA<br>GYVDGRGVIHRKRVGTFYGQASDDYREVNSGQEVGTYFIPGGCRAFGPGRINYFLNFWGPS<br>FSVDTACSSSLAAIQAACSSLWSGDIDMAITGGMNILSNSDVYAGLSQGHFLSPTGGCKTW<br>DEGADGYCRSDGVGSVVLKRLEDAEADNDNILAVVLSAATSHSAEAVSITHPHDAAQALL<br>YNQIVRRAGIDPLEVGYVEMHGTGTQAGDPTEMRSVTSVFAPPHIQGSRPIPLHVGSVKAN<br>MGHGEAAAGIMAFVKTMLVFQNGIIPPHIGVKTGLNPALPDLDKAGVVIPFRAANWRPTGT<br>KKRLAMVNNFGAAGGNTAMIIEEAKARPRLCEDIREAHAITISAKTAVSLSLNIKRLVEYIES<br>AQDLSLADVAYTVSARRRHYEYRKSVVVRSLAEAIKHLQPHIETAKSQTPTLVKRPPVAFA<br>FAGQGTFYVGIAAQIYRDSPFFRAQIDQFDNLARRQNFPSFLPAINKTCAHEDLPASSIHLAIV<br>CVEVALARMCMTFGIKPCAVIGHSLGEYAALAVAEVLSDSDTVFLVGTRATILESNCSPYTH<br>GMISVRASVDDISREADGLPFEVSCINGPNETVIGGTVENLEAVADRLSKVGYRKTRLDVPH<br>AYHTAQMDNVVNELIRQSQGIAYNTPKIPIMSPRDSSVIETGANIDSSYLPTSLKKAVDFAGA<br>LNAAWEAGVVSKSTVWLELSHHPVCSGFINRTLPNTSLTCSTLHRDSDNWTSLLKTLSSLYE<br>VGLNIDWNEYHRPFEHALRLVSAPTYAWNNKDYWIQYRGDWNLTKGQVLPEAELPAVSG<br>FRTSSIHRLYSENYDSSTAHLLGECNMTDLSLKGVIEGHAMNGYGVASSFLHAEMAFTLAR<br>RIQEKASLSTFTGMGINVTNFEYHDPVVKDASSLDPYPIVVDAEANLEMGEVQIKWFNPAIE<br>KWYCHAIAYYEDPSTWLSNWSRTTRLVTSRIDALVAMSNKGMANKLTTSLAYTLFGKLVD<br>YSSMYHTMQWVILNEDEAVAEVVFPADTQGDWAVPPHFIDGVVSLSGFILNGGTHFDNVN<br>NFFITPSWKSMRFAKPLAPGGRYLTYVRMIPEGVDDKGRLGSYVGDVYILQDGEIVGVVEA<br>ILFRQWPRIMLNRFFQPVGMAPPAPRVEKKRDAGRGTLPSSSSLQEKTTATAVTAKITARFPG<br>SVITPSRSAPISKSGSSPKIVPQLDYSLLTPRTSPNSDERIEKTDSDSGFEEADGANDVTSRAVEI<br>LAEELAVDKGLLTDECEIADIGVDSLMSLVISQKLREDLGIEVRDAFYLEVTTIGDLKKLLS

Table S3. *Cont.*

**>gi|297242555|gb|ADI24954.1| GsfB (*Penicillium aethiopicum*)**
MASNTSRSAHLAQIITENTANIETYRREQGLPPLSLGPDAPLDVKYPPNVEKCRRAVIDATLE LGELATGPVELRLVPGWAIMTMFGVTQFICDFDIARQIPLAGDISYEDLSKAINVAVPVLRQ VLRAGMPYHMFYESRPGHVAHTATTKVMASESLISDWTSLYTDVLFPASAGLSKALREEPT ASDPSKTGFMVTKGDGESGMYMYFEKHPEEARRFAGVMEAFQKDEAYAVRHLTDSWPSD SQTGKLVDLGGSTGAVAFALAEKFPGLEIVVQDLPGALEAAHVREGKNVSFMPHDFFNEQP VKDADVYMFRWILHNWPDGHVQRILRALVPSLKPGAKVIVFDEIMPPAGTLPLSIERYQRNI DFGMLTLFNSKIRDIVEWKEIITQSDQRFNVTGVRYPENSRLSLIEIVWQP
**>gi|297242556|gb|ADI24955.1| GsfC (*Penicillium aethiopicum*)**
MTLDQISRIQALVAEIADLSQTFEGPSERFMVDGLPFLRKSEELVTLVQSPAEHATSLIIKTME TAVIRTLLSLNVLQTIPTTGSITLQSLAVATETQESLLERLLRVVTKTGFIIRENGGYSHSHTSL AYAGPLGALFAPCYDEGIRALVRLPEYLSVKDKEEAKNARYSLFTWNEGQEGKATFEILST MPARTEGIHTLAMNVQHLRPYTGFFDYSKLVSEDRERPVFVDVGGGNGHVIKEILQAFPQIR PEQCVLEDRAETLELARTTGLLPAGVQLLEHDYLTRQPVSNAKAYHLRAVAYNLGDAELV QLLKQIVPVMGADSKVLIAENILFDDNSTVFSTVSDMIMLGIGGKERTEQNFREVLYEAGLTI EGIHRAPGLEYGIMEASLATS
**>gi|297242557|gb|ADI24956.1| GsfD (*Penicillium aethiopicum*)**
MSTPEQWIQEFEALCSRASILFPSGIEDENIRIRALRIAEKAVHQLHTPMTFAEAQTWAPLELF GAGVACEMGIFDVLSKSSVPLSPVDIATELNTDPALVARIMRLLDAHYMVDQVTLGQYAA NAITRDYVQPYRKGNVMTQVALMPSYFALPAWLRDNDYKVRPDANHCAWQVGANTTKT FWEMPRTTQEDDFVTFYPFEAVFSTSNVDDILFVDIGGGLGHQAMRVRSAFPRSRGRIIVQD LPQVTNKITTASLPDVEIMDHDMADPQPVKGARVYYLRGVLHNHADHISIKYLSQFAAAMS PESRLLIHEALATDLNPTKNITRFDLSMLASCGGAQRSEAEQKALLEKVGLEVSGVWSTPRD WSIMEARLKRE
**>gi|297242558|gb|ADI24957.1| GsfE (*Penicillium aethiopicum*)**
MPKTAFITGANGLSGSAIVEYLCNTTTSDDWGSIIVTSRSPFKSTVMDPRIKFIALDFVNDVSS LVETMKEVCGAVTHAYFCSYLHKDDFAESYTVNKALFENFIAAIDKAAPKLENVTLQTGG KYYNLHVEPVPSPARENDPRRYGPFENFYFTQEDTLAEMQRGKTWSWNVIRPEAIIGANSQ PYGLNVALTIAMYFLICRELGSASPMPTNQRYWEGTDDVSYAPLIADLTIFVSTRKSCANEA FNVTNGDYFTWRYMWPRLAASLGAKADSQQCFEKPMPGEGELQLDWSLAEWCKDKRKV WEDLCDRQGLPGAKATFDLAGWAVGDFLYQRTWSATLSVNKARRFGWTGHMDSYQSFV DTFDKFRQLGLIPK
**>gi|689554835|gb|ADI24958.2| GsfF (*Penicillium aethiopicum*)**
MTVLFILSAGLVAVFGYLVSWFIYCRTLHPLSKVPGPFWPSVTRLWLTYAVSRGELDVVQR DLHRRYGPLVRIAPDEIACADPEAIRKIYSTTSPLNKSDFYHIWDVGAFSKYPNAFAIVDENM HFERRRIVSSVYSMSTVLTLEPYIDNCSRLFVKRMTERTVPHEAIDLGDWFLWYAYDVIGEL FFGHSLGFIENRGDEGGFLASLEVMLPVLTIAAASSPLVRGLIMGLFTLSSTARKGLKGMNHI IETARASVDKRASAVAEPGKGERKDLLHNLLNIVSSKGDKLDFGIEDVKNEAFAALTAGAD ATMIELQAIFYYLVKDRSVYEELRKEVDQAVETGKLSEFPSYSEVVQLPLLKATIKEALRLH PAVGFTMPRVVGQAGIELLGMYIPPGWKVGMNAAVVGRDEGVYGTDANTFRPERWIERT DSDMDRCNNLVFGAGTRTCIGKQIALSEIYKMVPLLIRKFDFALVDPSKSWTTHDYFFNKQS GVQVKVTVRGL

Table S3. *Cont.*

**>gi|297242560|gb|ADI24959.1| GsfG (*Penicillium aethiopicum*)**
MLCSSCGVPTSTQDPSSPRAKAAAADRRREQNRRAQKRFRQKHREQKATPEQDQSQPQKR ASDAMDTVGPTTSKESTPLTPPSCYEERQADSVVDYDCLLDPSDRCVMNKWTDDFWTSTL EETAPFAATALVLPSERKDSQEILTMRDFDDSVDKNQNGRVPAGPTVLHRAVQTGNSKVV GLLLEHNANCNTKDNTGLTPLLCAVIGGHEEVLELLLSHGASIGHVDDAHWSALHWAVFH KRHRILERLLRCCSGDSSLLNIRNKDGETPLSVAVSAGSEVAVKLLLEFGATVNIEQSS
**>gi|297242561|gb|ADI24960.1| GsfH (*Penicillium aethiopicum*)**
MFISIGIKAVLLLVASIPAQAWNRLDKDNAALLIIDHQVGLAQVVRDYNTNDFRNNILGHAA LGNVFNLPTVLTTSSDAGPNGLMLKEITDMHPNATFVRRQGEVNAWDNADFRAAVEATGK KQLIIAGIVTEVCTSFLALSLVDAGYEVFANTDASGTFDARLAEDANRRMEKAGVTLMGLF GIVCDLMRDWRKTPGLPEVLPFLDKYQFGYGLVARHHAGAIQNGTFFPVEGALI
**>gi|111055548|gb|EAT76668.1| hypothetical protein SNOG_15830 (*Phaeosphaeria nodorum* SN15)**
MAYTPMEQPLQFSVTRIGQDLKIFEILTNAGPQSLQELQILTHAHPITLALSLTGRLLRYMSS VGLIHETGVNQFEASKKSQNLATPEAEKIVTHFFENCGPLFQEMPAFLRSKKYQDITDGQNT VFQPAYNTDLDTNEWFSQNPLHMGARIKYMAMEQAVRGRWLNVYPIQSQLKGWNPELPA FVDVGGSVGHYCTMFKNNFPDVPGRVILQDLPDTLSHALKTPGVELMGHDFFQPQPIKGAK FYHLGWILNNWNDDESKQILRQIRSAMNAESVVLINDVILPEAGVPSFAASLDLVMLGACG SRERMRKEWDEILADVGLIVKDCIVYHHELCHGIIGAKLA
**>gi|111055549|gb|EAT76669.1| hypothetical protein SNOG_15831 (*Phaeosphaeria nodorum* SN15)**
MAETIKLEVKDSAHRRRSVPLLSSGERQPPAKTRNGDCIVLPSKTPTSLASTIVDDVLVDCN GPSCTLMGKETRERLAIGQSDALLVMFYLDNLFPFLFPFYRPSLLEGGRTWILEMMICSPVV KQALLCQSSCFMSMAQGMANWEMVLEQTRDAFKVLRLSLQVISDAGVTEHIHGTVRILASI VQVQRFEVAVLSFDNCQAHLNVALSLFSQLLESTDDTNTACPSSSFNAVLNQLKPKTWSAS TGVFQVPIAEQAAFRFSSALLILDDIIAATILQEQPRLYNYHRSLLCTPNSSGAVVNLEETVGC QNWALLYIGEIATLDAWKQQYQHTGSLDLMELVRRATDIEALVEGHVLQLESELLGASSEG ASLLDMFDSDSRQTKTSASQITLVTRVWAHAALIYLSVVVSGWQPANLDVRYHVSQTFELL TQQIVPPALLRSMVWPFCVAGCMAAPEQEAQLRTMVQVLQPPSMFGTVRKALEIMEDVWS KRGMGDAGKRDLATCFKNQGDLVLLV
**>gi|160705606|gb|EAT76670.2| hypothetical protein SNOG_15832 (*Phaeosphaeria nodorum* SN15)**
MLPISSYISRLSCFGTKPKPKPKPIKSRISKLSTQTCFTITKDTNMATVVLILGSGPRVGASVA QTFKESGYSVALASRKGSNSVTDDGYFSIKADLASPASILGVFSTIKSKFGAAPSVVIYNAAA LTPPSDPSSVLSVSTEAVTKDLNINVVSPYVAAQQAIAAWETLPQDAKKTFIYTGNVLNELI VPIPMFLTLGIGKAASAYWVGLADTLYKARGYRFFHADERHPDGKNKGMALDGPAHGEY YHELAQHPDGVPWQATFVKGQGYRSFK
**>AN7070|pkgB COORDS:ChrIV_*A_nidulans*_FGSC_A4:1131258-1129968C, translated using codon table 1 (340 amino acids)**
MSGGFYSSPFWAGYLETQRSRLPVLPEIDDGLSHCVVRFLGYNPGSMQLQGTNTYLVGTGS TRILIDTGEGAPQWAVSVTRYLEDHDISISHVLLTHWHKDHTGGVADLLAHDPSIIVYKHAP DPGQQAIANGQTFKTQGATLRAVLTPGHAVDHMCFLLEEENALFTGDNVLGHGYSVAEDL ETYTASLRLMAGLKCSVGYPGHGDAILNLPQTIARYISQRVAREKKIYAILALHACSCSSRN GGSTSSIGSVSESGDSDEEDNNMKTSRPAMQGLSTAEIGGLVYGESVKNSPTFDSAVGPLLN QVLYMLLEQGKCCDHVSILVIFQKPGFFSIPVI

Table S3. *Cont.*

**>AN7073 COORDS:ChrIV_*A_nidulans*_FGSC_A4:1120517-1119359C, translated using codon table 1 (321 amino acids)**
MEAYKTQPPVRKLKDSCDVCSASKLRCDKQKPTCARCANLNRPCTYSPARRGGRPHRVRR DRSKSQSQSQSSRQFFGMPDANTSSSPFAEPTRVPSQTDRGMSCSNDWFLRTQSRVHDHIQD SQPSAQSAKVSPSPCKNPMNTRLAAETAETDMDCTRVALSIVEQLERSQEQRPRSTAPTYTH GGLTATEACQRLLTILMCPCSDQAEVALLVASGCISLMDVIHSSAGFASESLGHDGSVSSCN SPPISSEQDPLIRSWSQPQSISRSCSLASDSRSQVGDLSKIAKVIVQFTERYCQDAKVAAEPRA HWVTRYESLSR
**>AN7072 COORDS:ChrIV_*A_nidulans*_FGSC_A4:1121945-1122955W, translated using codon table 1 (336 amino acids)**
MELPAESELQYAGECLSLPGTFLEPPIEDPPSSVLNLLNLSQVDFNSYDFSSLGSREFSSKWQ TNTPLCTDSLSDESAPGLLTEDMGISPIPMPAAEATCPQESEDRLCRNPQGRCISLATGILGSM HAGSNSCILQVATSDQGGASDRQPQQSRAADAILSMNQSALRTVRSILNCSCYESPQVLLLV TVMCSRITAWYWRIADIYSYSHGNPTAGSPRAALPTSVGSRAETRRRDFFIGNHRLDREVET VVIRHVLLGMLQELQLVIRDFAGQAGQSPAGTVDTDDPTSTSDLMLSGMRARVVAFLRKQ LHSLTSALDHTDSGFGTMGPHVSHY
**>AN10884 COORDS:ChrIV_*A_nidulans*_FGSC_A4:1131717-1134001W, translated using codon table 1 (647 amino acids)**
MMNVPEKCKVLVVGGGPAGSYAASALAREGIDVVLLEAEKFPRYHIGESMLPSMRHFLKFI DAYDKWDAHGFNIKKGGAFRLNWSRPETYTDFIAAGGPGGYAWNVIRSEADELLFKHAAE CGVKTFDETKVASIEFSSPDLSSGGTHPFGRPVSATWTRKDGTSGTISMDYIVDASGRNGLIS TKYLKNRSYNKGLKNVASWGYWRGGGVHGVGTHKEGAPYFEALKDASGWVWFIPLHNG THSVGVVQNQEMATEKKRKMAEPSSKGFYLESLEFVPGIKELLANAELISEVKSASDWSYS ASSYAFPGVRIAGDAGSFIDPFFSSGVHLALSGGLSAATTIAAAIRGDCDENVAASWHDKKT SESYTRFLLVVSSALKQIRSQDEPVISDFDEGSFERAFDLFRPIIQGQADADAKGKLTQAEISK TVEFCFRAFAHVSFEQKEALVQKLKSLGHDGDAYDENNRKALEEIEKQLTPEEQTILKTLKG RRMVRPEDSLNIDNFTLDSIDGLAPRLEKGKLGLSAAKKAELKFTAHDPLSFLNGEAMAAQ KTSPNGNLEINGHTQTNGNHLANGHGEVNGHSNAGASSAKSCMADLIAAENDSSQPSFDEA TRHRLISSLQQSAEELETPYDTVLRYVNAVSQIN
**>AN10889 COORDS:ChrIV_*A_nidulans*_FGSC_A4:1134037-1135110W, translated using codon table 1 (337 amino acids)**
MLTALSKQGRQTALVCIGGDLGIFKSLAESKAPLSSKQLAEATMADPLLVSRIMRYLVASRL VGETAPDQYVATKKTYVFADPRFEEPIRFFHAVSNRAFQALPEFLKETGYQNETQRSAFQK GLGTELQLYPWLKQHPDVLKNFQAAMRLTKDANGVGVMPLDSSVSSGHEGVMFVDIGGN TGHQAAEVLSQHPELAGRVTVQDRGEVIKSAPEMKGIQWMEHDFFDVQPVKGAKYYYLR AILHNWDDDHAVQILANIVPAMSADSLVAIDEVVVPDRDAHLWPAGLDLQMYTIFGTRER TAAQWDAILDRAGLRAVAVKRYAPVMQSSVIFAAAK

Figure S1

Figure S1. A maximum likelihood phylogenetic tree constructed with FastTree [1] from the KS domains of the 908 PKSs used in this study. Bootstrap values are shown next to the internal nodes of the tree. The clade corresponding to the 188 group V PKSs is highlighted in green. The leaves of the tree are labeled with the accession number of the PKS and its corresponding species.

Figure S2

Figure S2. A maximum likelihood phylogenetic tree constructed with FastTree [1] from the KS domains of the 188 group V PKSs extracted from the larger set used above. The bootstrap values are presented next to their corresponding nodes and the leaves are labeled with the accession number of the PKS and the corresponding species. The characterized PKSs belonging to groups V1, V2, and V3 are shaded in blue, green, and yellow, respectively [2–15]. These shaded boxes include closely related PKSs from the same species in which the characterized cluster was originally described. Next to the tree are the gene clusters corresponding to the PKSs that were identifiable through MultiGeneBLAST analysis. Gene cluster diagrams next to brackets depict the cluster corresponding to the PKS with its accession number highlighted in red, but all of the bracketed PKSs belong to clusters which are identical in terms of the presence and synteny of their group V-cluster homologs. Genes are represented as arrows with a color corresponding to the proteins they encode which are detailed in the color key to the right of the cluster diagrams. These color keys are subgroup-specific, and black lines extending from the tree to the color keys delimit the subgroups. Genes with no color were not identified as homologous to any group V1 cluster gene. Colorless genes that were noted as occurring frequently in related clusters are labeled adjacently. The products of the characterized clusters are shown at right. Group V1: PKS = Polyketide synthase, MβL = Metallo-β-lactamase-type thioesterase, AO = Anthrone oxidase, OGO = 2-oxoglutarate-Fe(II)-type oxidoreductase, EthD = EthD domain-containing protein, a putative decarboxylase [3,7], MCO = multicopper oxidase, P450 = cytochrome P450, SMT = S-adenosylmethionine-dependent methyltransferase, BVO = Baeyer-Villiger oxidase, DOR = Pyridine nucleotide-disulfide oxidoreductase, FDH = Flavin-dependent halogenase, AflS = Transcriptional co-regulator of the aflatoxin biosynthetic gene cluster [16], AflR = Transcriptional regulator of the aflatoxin biosynthetic gene cluster, C6TF = GAL4-like $Zn(II)_2Cys_6$-domain and fungal-specific transcription factor domain-containing protein, NOR = NADH-dependent oxidoreductase, OMT = *O*-methyltransferase, GST = Glutathione *S*-transferase, MT = Methyltransferase, SD = Scytalone dehydratase, KR = Ver-1-like ketoreductase [4,17], FMO = Flavin-dependent monooxygenase, ACS = Acyl-CoA synthase, Kelch = Kelch domain-containing protein, SDR = Short-chain dehydrogenase, GA4D = GA4 desaturase family protein, MFS = Major Facilitator Superfamily transporter, GMCOR = Glucose-methanol-choline oxidoreductase, *vbs*-like, MFS = Major Facilitator Superfamily transporter, PPT = Polycyclic prenyltransferase, AKOR = Aldo/keto

oxidoreductase, SH = Salicylate hydroxylase. Group V2: C6TF = GAL4-like Zn(II)$_2$Cys$_6$-domain and fungal-specific transcription factor domain-containing protein, VrtR1-like, MFS = Major Facilitator Superfamily transporter, P450 = Cytochrome P450, TA = Threonine aldolase, OGO = 2-oxoglutarate-Fe(II)-type oxidoreductase, PKS = Polyketide synthase, ACS = Acetoacetyl-CoA synthase, PPT = Polycyclic prenyltransferase, IDS = Isoprenyl diphosphate synthase, MT = Methyltransferase, MβL = Metallo-β-lactamase-type thioesterase, FMO = Flavin-dependent monooxygenase, TF = GAL4-like Zn(II)$_2$Cys$_6$-domain and fungal-specific transcription factor domain-containing protein, VrtR2-like, NDH = NAD-dependent dehydratase, LIP = Secretory lipase, ART = Arrestin, CNR = Copper-containing nitrite reductase, MGT = Magnesium transporter, ABCT = ABC transporter, Abr2 = Conidial pigment laccase, OMT = *O*-methyltransferase, EthD = EthD domain-containing protein, putative decarboxylase [3,7], PksP = Conidial pigment polyketide synthase. Group V3: C6TF = GAL4-like Zn(II)$_2$Cys$_6$-domain and fungal-specific transcription factor domain-containing protein, GsfR2-like, SDH = Short chain dehydrogenase, TF = GAL4-like Zn(II)$_2$Cys$_6$-domain and fungal-specific transcription factor domain-containing protein, GsfR1-like, DRT = Drug resistance transporter, EmrB subfamily, FDH = Flavin-dependent halogenase, PKS = Polyketide synthase, OMT = *O*-methyltransferase, NSD = Nucleoside-diphosphate-sugar dehydratase, P450 = Cytochrome P450, ANK = Ankyrin repeat-containing protein, AHD = YcaC-related amidohydrolase, FDH/OMT = Flavin-dependent halogenase and *O*-methyltransferase bifunctional protein, MβL = Metallo-β-lactamase-type thioesterase.

References

1. Price, M.N.; Dehal, P.S.; Arkin, A.P. Fasttree 2-approximately maximum-likelihood trees for large alignments. *PLoS ONE* **2010**, *5*, e9490.
2. Lim, F.Y.; Hou, Y.; Chen, Y.; Oh, J.H.; Lee, I.; Bugni, T.S.; Keller, N.P. Genome-based cluster deletion reveals an endocrocin biosynthetic pathway in *Aspergillus fumigatus*. *Appl. Environ. Microbiol.* **2012**, *78*, 4117–4125.
3. Chiang, Y.M.; Szewczyk, E.; Davidson, A.D.; Entwistle, R.; Keller, N.P.; Wang, C.C.; Oakley, B.R. Characterization of the *Aspergillus nidulans* monodictyphenone gene cluster. *Appl. Environ. Microbiol.* **2010**, *76*, 2067–2074.
4. Simpson, T.J. Genetic and biosynthetic studies of the fungal prenylated xanthone shamixanthone and related metabolites in *Aspergillus* spp. Revisited. *Chembiochem* **2012**, *13*, 1680–1688.
5. Nielsen, M.T.; Nielsen, J.B.; Anyaogu, D.C.; Holm, D.K.; Nielsen, K.F.; Larsen, T.O.; Mortensen, U.H. Heterologous reconstitution of the intact geodin gene cluster in *Aspergillus nidulans* through a simple and versatile PCR based approach. *PLoS ONE* **2013**, *8*, e72871.
6. Xu, X.; Liu, L.; Zhang, F.; Wang, W.; Li, J.; Guo, L.; Che, Y.; Liu, G. Identification of the first diphenyl ether gene cluster for pestheic acid biosynthesis in plant endophyte *Pestalotiopsis fici*. *Chembiochem* **2014**, *15*, 284–292.

7. Throckmorton, K.; Lim, F.Y.; Kontoyiannis, D.; Zheng, W.; Keller, N.P. Redundant synthesis of a conidial polyketide by two distinct secondary metabolite clusters in *Aspergillus fumigatus*. *Environ. Microbiol.* **2015**, doi:10.1111/1462-2920.13007.
8. Chooi, Y.H.; Fang, J.; Liu, H.; Filler, S.G.; Wang, P.; Tang, Y. Genome mining of a prenylated and immunosuppressive polyketide from pathogenic fungi. *Org. Lett.* **2013**, *15*, 780–783.
9. König, C.C.; Scherlach, K.; Schroeckh, V.; Horn, F.; Nietzsche, S.; Brakhage, A.A.; Hertweck, C. Bacterium induces cryptic meroterpenoid pathway in the pathogenic fungus *Aspergillus fumigatus*. *Chembiochem* **2013**, *14*, 938–942.
10. Chooi, Y.H.; Cacho, R.; Tang, Y. Identification of the viridicatumtoxin and griseofulvin gene clusters from *Penicillium aethiopicum*. *Chem. Biol.* **2010**, *17*, 483–494.
11. Ahuja, M.; Chiang, Y.M.; Chang, S.L.; Praseuth, M.B.; Entwistle, R.; Sanchez, J.F.; Lo, H.C.; Yeh, H.H.; Oakley, B.R.; Wang, C.C. Illuminating the diversity of aromatic polyketide synthases in *Aspergillus nidulans*. *J. Am. Chem. Soc.* **2012**, *134*, 8212–8221.
12. Chooi, Y.H.; Muria-Gonzalez, J.M.; Mead, O.L.; Solomon, P.S. *SnPKS19* encodes the polyketide synthase for alternariol mycotoxin biosynthesis in the wheat pathogen *Parastagonospora nodorum*. *Appl. Environ. Microbiol.* **2015**, doi:10.1128/AEM.00278-15.
13. Saha, D.; Fetzner, R.; Burkhardt, B.; Podlech, J.; Metzler, M.; Dang, H.; Lawrence, C.; Fischer, R. Identification of a polyketide synthase required for alternariol (AOH) and alternariol-9-methyl ether (AME) formation in *Alternaria alternata*. *PLoS ONE* **2012**, *7*, e40564.
14. Szewczyk, E.; Chiang, Y.M.; Oakley, C.E.; Davidson, A.D.; Wang, C.C.; Oakley, B.R. Identification and characterization of the asperthecin gene cluster of *Aspergillus nidulans*. *Appl. Environ. Microbiol.* **2008**, *74*, 7607–7612.
15. Li, Y.; Chooi, Y.H.; Sheng, Y.; Valentine, J.S.; Tang, Y. Comparative characterization of fungal anthracenone and naphthacenedione biosynthetic pathways reveals an α-hydroxylation-dependent claisen-like cyclization catalyzed by a dimanganese thioesterase. *J. Am. Chem. Soc.* **2011**, *133*, 15773–15785.
16. Ehrlich, K.C.; Mack, B.M.; Wei, Q.; Li, P.; Roze, L.V.; Dazzo, F.; Cary, J.W.; Bhatnagar, D.; Linz, J.E. Association with AflR in endosomes reveals new functions for AflJ in aflatoxin biosynthesis. *Toxins* **2012**, *4*, 1582–1600.
17. Skory, C.D.; Chang, P.K.; Cary, J.; Linz, J.E. Isolation and characterization of a gene from *Aspergillus parasiticus* associated with the conversion of versicolorin A to sterigmatocystin in aflatoxin biosynthesis. *Appl. Environ. Microbiol.* **1992**, *58*, 3527–3537.
